# Mechanistic Exploration of Yiqi Zengmian in Regulating the Microenvironment as an Immunopotentiator with the Beijing Bio-Institute of Biological Products Coronavirus Vaccine Based on Transcriptomics and Integrated Serum Pharmacochemistry

**DOI:** 10.3390/ph18060802

**Published:** 2025-05-27

**Authors:** Zeyue Yu, Yudong Wang, Jianhui Sun, Xiaotong Zheng, Liyu Hao, Yurong Deng, Jianliang Li, Zongyuan Li, Zhongchao Shan, Weidong Li, Yuling Qiao, Ruili Huo, Yibai Xiong, Hairu Huo, Hui Li, Longfei Lin, Hanhui Huang, Guimin Liu, Aoao Wang, Hongmei Li, Luqi Huang

**Affiliations:** 1 National Resource Center for Chinese Materia Medica, China Academy of Chinese Medical Sciences, No. 16 Nanxiaojie, Dongzhimennei Ave., Beijing 100700, China; yy_015zy@163.com; 2 Institute of Chinese Materia Medica, China Academy of Chinese Medical Sciences, No. 16 Nanxiaojie, Dongzhimennei Ave., Beijing 100700, China; wydzzubio@163.com (Y.W.); sjh121858@sina.com (J.S.); haoliyu@hotmail.com (L.H.); dengyurong1999@163.com (Y.D.); jianliang0320@126.com (J.L.); lizongyuan0921@163.com (Z.L.); shanzc1996@163.com (Z.S.); hrhuo@icmm.ac.cn (H.H.); lihuiyiren@163.com (H.L.); linlongfei0417@126.com (L.L.); huanghanhui113@163.com (H.H.); 20190935101@bucm.edu.cn (G.L.); aoaowang@163.com (A.W.); 3 Beijing Institute of Biological Products Company Limited, No. 9 Boxing Second Road, Beijing 100176, China; zhengxiaotong20@sina.com (X.Z.); liweidong@163.com (W.L.); lilian0601@163.com (Y.Q.); 4 China Academy of Chinese Medical Sciences, No. 16 Nanxiaojie, Dongzhimennei Ave., Beijing 100700, China; huoruili@sina.cn (R.H.); yibaixiong@126.com (Y.X.)

**Keywords:** Yiqi Zengmian decoction, BBIBP-CorV, humoral immunity, cellular immunity, transcriptome analysis, network pharmacology

## Abstract

**Background**: Yiqi Zengmian (YQZM) functions as an immunopotentiator by enhancing both cellular and humoral immunity. However, its pharmacodynamic active constituents, particularly those absorbed into the bloodstream, and mechanism of action remain unclear. This study aimed to investigate the immunopotentiating effects and mechanisms of YQZM in mice immunized with the BBIBP-CorV (Beijing Bio-Institute of Biological Products Coronavirus Vaccine). **Methods**: Serum pharmacochemistry and ultra-performance liquid chromatography–tandem mass spectrometry (UPLC-MS/MS) were employed to identify bioavailable components of YQZM. The mice received the BBIBP-CorV twice on days 1 and 14, while YQZM was orally administered for 28 days. Neutralization assays and ELISA quantified antigen-specific antibodies (abs), flow cytometry (FC) and intracellular cytokine staining (ICS) were used to assess immune cell populations and their cytokines, and an enzyme-linked immunospot assay (ELISpot) quantified memory T and B cells (MBs and MTs). To identify underlying mechanisms, network pharmacology, RNA sequencing (RNA-Seq), molecular docking, Western blotting (WB), and quantitative reverse transcription PCR (RT-qPCR) were performed. **Results**: YQZM significantly enhanced antigen-specific antibody titers, immune cell proportions, cytokine levels, and memory lymphocyte functions. UPLC-MS/MS analysis identified 31 bioactive compounds in YQZM. KEGG enrichment analysis based on RNA-Seq and network pharmacology implicated the TLR-JAK-STAT signaling pathway in YQZM’s immune-enhancing effects. WB and RT-PCR validated that YQZM upregulated the expression of critical nodes in the TLR-JAK-STAT signaling pathway. Furthermore, molecular docking indicated that YQZM’s primary active components exhibited strong binding affinity for critical proteins. **Conclusions**: YQZM effectively enhances vaccine-induced innate and adaptive immunity via a multi-component, multi-target mechanism, among which the TLR-JAK-STAT signaling pathway is a validated molecular target.

## 1. Introduction

Over the past few years, the emergence of SARS-CoV-2 (severe acute respiratory syndrome coronavirus 2) has triggered a global pandemic, resulting in significant public health challenges and socioeconomic disruptions [1]. Although vaccines such as inactivated BBIBP-CorV have demonstrated robust immunogenicity and safety in clinical trials, efforts to control the pandemic face persistent challenges owing to the virus’s high transmissibility and evolutionary adaptability [2,3,4,5,6]. Key virological features, including immune evasion, rapid mutations in structural proteins, and seasonal fitness variations, undermine the efficacy of neutralizing antibody (NAb) responses and hinder durable population immunity [7,8]. Notably, immunocompromised patients, such as cancer patients, transplant recipients, and individuals with autoimmune disorders, exhibit significantly reduced NAb titers following BBIBP-CorV vaccination compared to healthy individuals. Similar deficits have been observed in elderly, pediatric, and comorbid populations [9,10,11]. These suboptimal immunization outcomes highlight the critical need to develop targeted vaccination approaches for immunocompromised populations. Consequently, investigating effective immunopotentiators—agents capable of enhancing vaccine-induced immune responses—has become a crucial research priority to bridge this protection gap.

Potential strategies in enhancing vaccine efficacy include dose escalation, increased vaccination frequency, alternative routes of administration, and the incorporation of adjuvants or immunopotentiators [12,13]. For immunocompromised individuals, the ideal immunopotentiator should be both effective and well-tolerated. Traditional Chinese Medicine (TCM) has garnered heightened acknowledgment in recent years as an immunopotentiator, owing to its historical role in enhancing host defense mechanisms and regulating immune functions [14,15]. TCM’s multi-component and multi-target nature may offer synergistic effects with vaccines, particularly for tailored immunological responses and mitigation of side effects. 

YQZM, a well-tolerated and nontoxic TCM compound formula, consists of six herbal components: Panax ginseng (Renshen), Poria cocos (Fuling), Astragalus mem-branaceus (Huangqi), Dioscorea opposite (Shanyao), Citri Reticulatae Pericarpium (Chenpi), and Glycyrrhiza glabra (Gancao). This formula is designed based on the TCM principle of “Fu Zheng Gu Ben” (strengthening vital energy and root immunity), which aligns with its observed immunomodulatory effects. Numerous studies have documented the immunomodulatory properties of bioactive constituents from botanical drugs. For instance, the active ingredients of Panax ginseng, like ginsenoside, act as vaccine adjuvants by enhancing dendritic cell (DC) maturation and Th1/Th2-balanced (T helper cell 1/T helper cell 2) responses [16,17]. Astragalus polysaccharides and Astragaloside promote the natural killer cell (NK) function and upregulate Toll-like receptor (TLR) [18,19]. Glycyrrhiza glabra flavonoids exhibit anti-inflammatory effects [20]. Previous research showed that YQZM could enhance both adaptive and innate immunity in healthy mice at high and medium doses (1.690 and 0.845 g/kg, respectively) (Supplementary Material S4), highlighting its potential as an immunopotentiator. These findings indicated that YQZM regulates immunity through multiple biological mechanisms. However, the complex and diverse composition of YQZM presents challenges in identifying the active constituents that mediate its therapeutic effects. Furthermore, the effect of YQZM as an immunopotentiator in vaccine-immunized mice remains unknown.

The integration of serum pharmacochemistry with UPLC-MS/MS has emerged as a viable approach for identifying the bioactive components in TCMs. Bioactive components are chemicals that are absorbed and circulate in the bloodstream after oral administration [21]. Similarly, network pharmacology has emerged as a powerful tool for identifying potential therapeutic mechanisms and elucidating the intricate network relationships between TCM and diseases [22]. RNA-seq complements this approach by elucidating TCM-regulated signaling pathways and therapeutic targets via transcriptome analysis. Collectively, these findings provide a comprehensive understanding of the holistic effects of TCM.

In this study, the immunoenhancing effects and underlying mechanisms of YQZM in a BBIBP-CorV-immunized mouse model were evaluated. Antigen-specific abs titers, proportions of immune cells and their cytokines, and the quantities of effective memory T and B cells were evaluated to assess the degree of the immune response. Additionally, serum pharmacochemistry, transcriptomics, RT-qPCR, WB, and molecular docking were performed to identify the specific bioactive components and elucidate the mechanisms of action. This integrated multimodal approach systematically characterized YQZM’s immunopotentiation mechanisms and established an evidence-based foundation for its development as a novel immunomodulatory adjuvant.

## 2. Results

### 2.1. Identification of Absorbed Components of YQZM in Rat Serum

**Figure 1:**
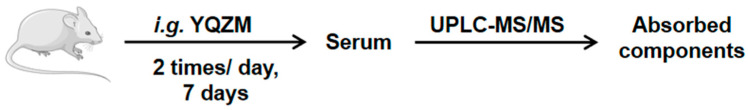
Experimental protocol for the preparation of control serum and serum containing YQZM.

**Figure 2:**
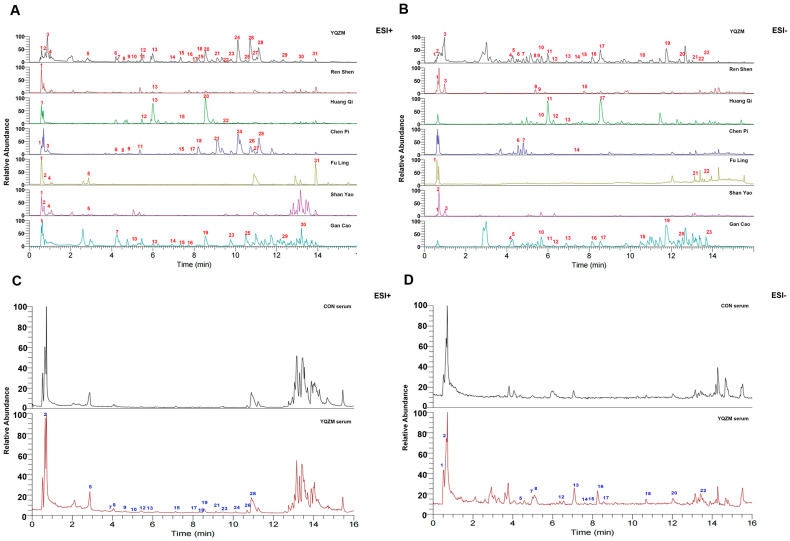
Chromatograms of YQZM extract and serum detected by UPLC-MS/MS. The *x*-axis represents retention time, and the *y*-axis represents the relative abundance of compounds. (**A**) Total ion chromatogram (TIC) of YQZM and single botanical drug (ESI+). (**B**) TIC of YQZM and single botanical drug (ESI−). (**C**) TIC of control serum and serum containing YQZM (ESI+). (**D**) TIC of control serum and serum containing YQZM (ESI−).

UPLC-MS/MS was used to analyze YQZM, individual botanical extracts, and serum containing YQZM which prepared as Figure 1. The ion chromatograms in the positive and negative ion models for YQZM, single-drug extracts, control serum, and serum containing YQZM are shown in Figure 2A–D. A total of 52 compounds were identified in YQZM (31 in positive ion mode and 23 in negative ion mode), including flavonoids, terpenoids, amino acids, steroids, and their derivatives. The residence time (RT), molecular formula, and identification of material components are provided in Table 1 (ESI+) and Table 2 (ESI−). Additionally, 31 components were identified in the serum containing YQZM and are listed in Table 3 (ESI+) and Table 4 (ESI−). The components found in the blood were the main and possible contributors to the therapeutic effects of the drug, suggesting that YQZM might boost immunity through 31 absorbed components.

### 2.2. YQZM Increased Immune Organs Index in Immunized Mice

**Figure 3:**
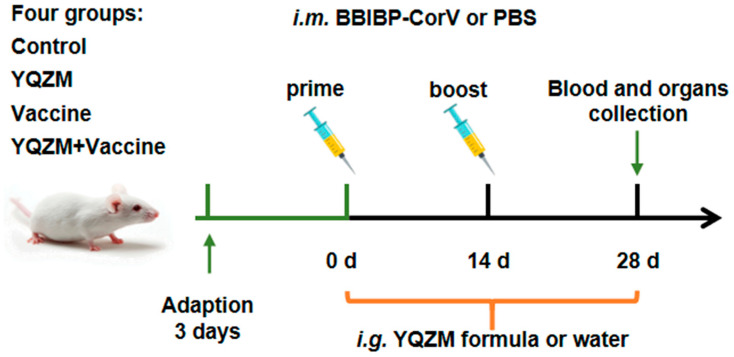
Experimental protocol for the immunization of mice with the BBIBP-CorV and the treatment schedule with YQZM.

**Figure 4:**
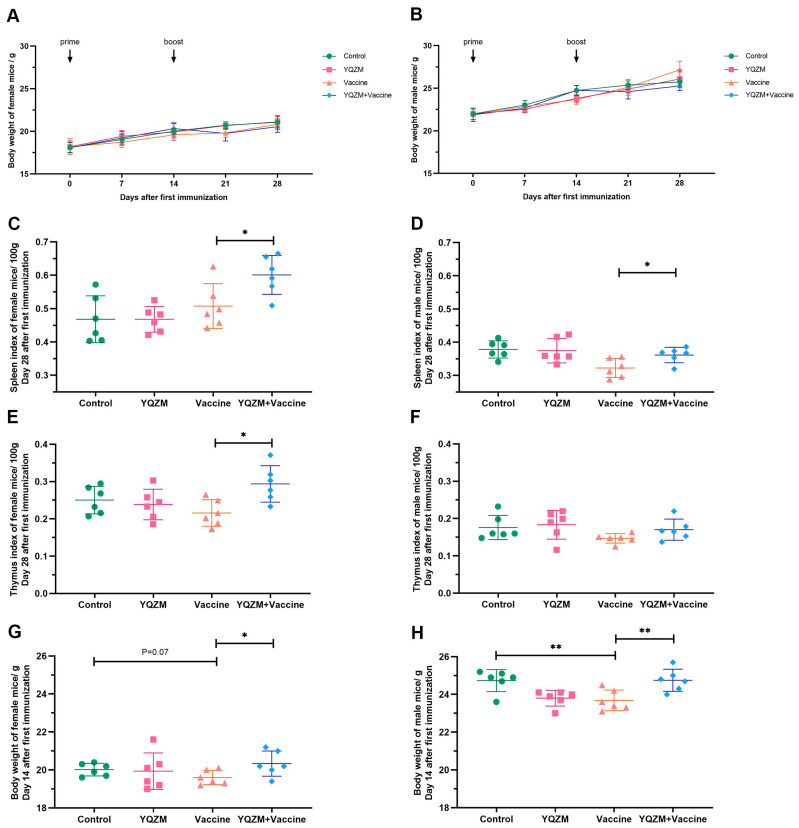
The immune organ index and body weight of immune mice with BBIBP-CorV. (**A**,**B**) The body weight change of female and male mice over 28 days following the administration of YQZM. (**C**,**D**) The spleen index (mg/100 g body weight) of female and male mice. (**E**,**F**) The thymus index (mg/100 g body weight) of female and male mice. (**G**,**H**) The body weight of female and male mice over 14 days following the administration of YQZM. * *p* < 0.05, ** *p* < 0.01.

The immune mouse model was prepare as Figure 3. Changes in the body weight of female and male mice following administration are shown in Figure 4A,B. The body weights of the YQZM+ Vaccine group were significantly higher than those of the Vaccine group by day 14, although the final body weights did not vary significantly (Figure 4G,H). Furthermore, the splenic index in the YQZM+ Vaccine group was much greater than that in the Vaccine group (Figure 4C,D). The thymus index of female mice markedly increased following YQZM administration, whereas no significant changes were observed in male mice (Figure 4E,F). These results suggested that YQZM enhanced immune organ indices in immunized mice.

### 2.3. YQZM Induced High Spike-Specific Antibody

**Figure 5:**
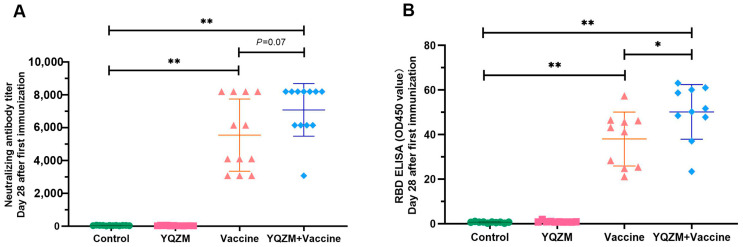
Antigen-specific antibody titers on immune mice with BBIBP-CorV. (**A**) Nabs titers in serum of immune mice with BBIBP-CorV over 28 days following administration of YQZM. Neutralization test used fixed virus-diluted anti-serum method. (**B**) Anti-RBD antibody levels in serum of immune mice with BBIBP-CorV over 28 days following administration of YQZM via ELISA test. * *p* < 0.05, ** *p* < 0.01.

To assess the efficacy of YQZM on antigen-specific Abs, we measured receptor-binding domain (RBD)-specific Abs and NAbs in the serum using ELISA and neutralization assays. As shown in Figure 5A,B, the RBD-specific antibody titers were significantly higher in mice treated with YQZM and the BBIBP-CorV than in those treated with the BBIBP-CorV alone. Additionally, the geometric mean titers (GMTs) of NAbs against SARS-CoV-2 were elevated in the YQZM+ Vaccine group compared to those in the Vaccine group. The NAb titers were 6631 (95% CI = 6348, 6913) in the YQZM+ Vaccine group, versus 5313 (95% CI = 4977, 5653) in the Vaccine group. These results demonstrate that YQZM significantly enhanced spike-specific antibody production in BBIBP-CorV-immunized mice.

### 2.4. YQZM Improved Proportion of Innate Immune Cell Subsets

**Figure 6:**
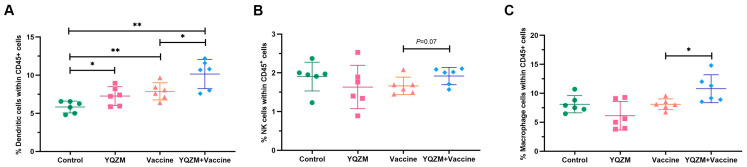
Innate immune cell subsets on immune mice with BBIBP-CorV. (**A**) Percentage of DCs in mice spleen (CD45^+^ CD11c^+^ MHC-II^+^). (**B**) Percentage of NK cells in mice spleen (CD45^+^ CD49b^+^ CD3^−^). (**C**) Percentage of Mø in mice spleen (CD45^+^ CD11b^+^ F4/80^+^). * *p* < 0.05, ** *p* < 0.01.

Changes in innate immune cells in the immunized mice were investigated using FC. Single spleen cells were isolated, stained, and labeled with surface fluorescent antibodies. The findings revealed that YQZM significantly increased the proportion of natural killer (NK) cells, dendritic cells, and macrophages (Møs), suggesting that YQZM could promote the innate immunity response by increasing the proportion of DC, NK, and Mø cells (Figure 6A–C, Supplementary Material S1).

### 2.5. YQZM Enhanced the Cytokine Secretion of T Cells and Memory T Cells

**Figure 7:**
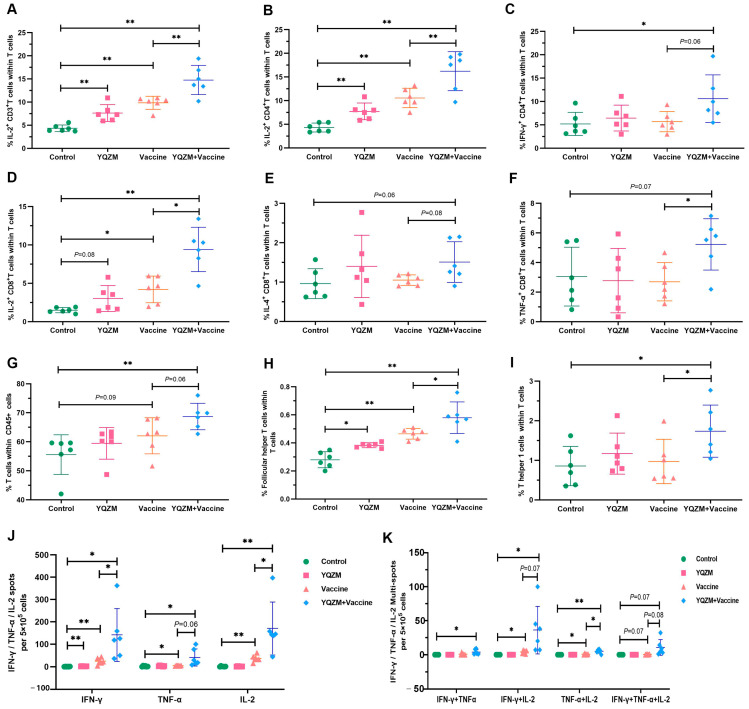
Subsets of T cells and their cytokine in immune mice with BBIBP-CorV. Antigen-specific T cytokine production analysis in T cells by flow cytometry. Cells were stained with anti-CD3 plus anti-IL-2 (**A**), triple–stained with anti-CD3 plus anti-CD4 plus anti-IL-2 (**B**) plus anti--IFN-γ (**C**), or triple-stained anti-CD3 plus anti-CD8 plus anti-IL-2 (**D**) plus anti-IL-4 (**E**) plus anti--TNF-α (**F**). Percentages of T cells in CD45^+^ cells (CD45^+^ CD3^+^), Tfh cells (CD3^+^ CD44^+^ CXCR5^+^), and Th1 cells (CD3^+^ CD44^+^ CXCR3^+^) in total T cells are shown in (**G**), (**H**), and (**I**), respectively. Results of ELISpot of S-specific IL-2, IFN-γ, and TNF-α for memory T cells are shown in (**J**), and multiple fluorescent spots are shown in (**K**). * *p* < 0.05, ** *p* < 0.01.

ELISpot and FC were used to assess SARS-CoV-2-specific immune response [23,24]. ICS and FC were used to analyze cytokine expression in CD4^+^ and CD8^+^ T cells to assess the impact of YQZM on the S-specific T cell response. As shown in Figure 7A–F and Supplementary Material S2, the secretion of IL-2 in S-specific CD3^+^ T cells; IL-2 and IFN-γ in S-specific CD4^+^ T cells; and IL-2, IL-4, and TNF-α in S-specific CD8^+^ T cells were dramatically enhanced in the YQZM+Vaccine group. Moreover, the proportion of T cells, Tfh cells (follicular helper T cell), and Th1 cells were significantly increased in mice receiving both YQZM and the vaccine (Figure 7G–I, Supplementary Material S2). The cytokine profile of the immune response is an important indicator of Th-cell bias. These findings showed that YQZM, as an immunopotentiator, enhanced both Th1 and Th2 responses and the activities of CD8^+^ T cells. To further determine the actual immune status and ability of memory T cells, the S-specific cytotoxic response was tested in vivo using ELISpot. The finding showed that S-specific IL-2, IFN-γ, and TNF-α of T cells in mice immunized with the vaccine and YQZM reached 171, 142, and 41 (per 5 × 10^5^ cells), respectively, whereas they were 37, 25, and 5 (per 5 × 10^5^ cells) in the vaccine-alone group (Figure 7J,K, Supplementary Material S2). These data suggest that YQZM serves as an effective immunopotentiator by increasing S-specific memory T activity.

### 2.6. YQZM Raised the Immunoglobulin Secretion of Memory B Cells

**Figure 8:**
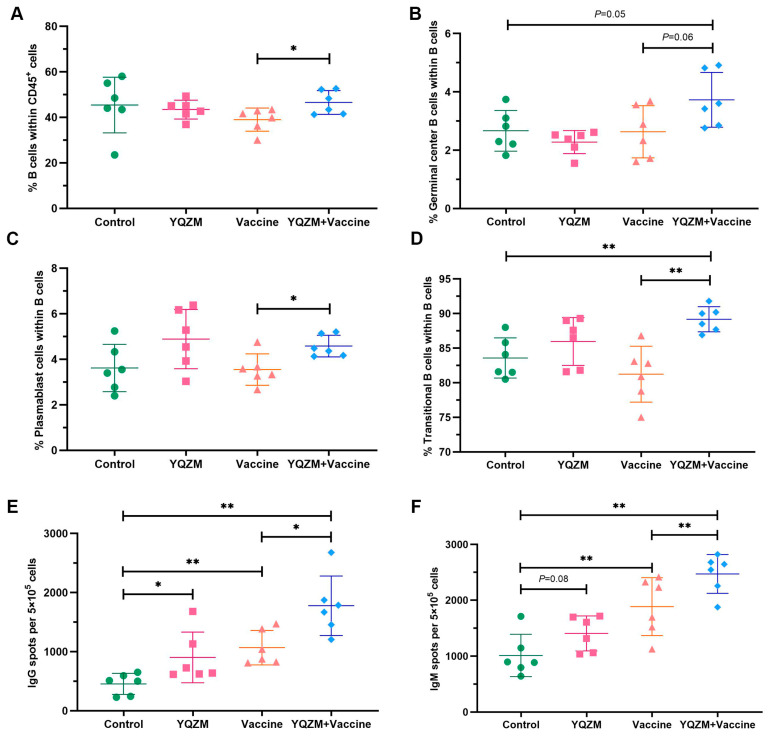
The subsets of B cells and their immunoglobulins in immune mice with the BBIBP-CorV. The percentage of B cells (CD45^+^ CD19^+^), germinal center B cells (Fas^+^ GL7^+^), plasmablast cells (CD138^+^ B220^+^), and transitional B cells (CD19^+^ CD40^−^) are shown in (**A**), (**B**), (**C**), and (**D**), respectively. The results of ELISpot of S-specific IgG and IgM of memory B cells are shown in (**E**,**F**). * *p* < 0.05, ** *p* < 0.01.

Antibody titers can be maintained by the presence of long-lived plasma cells or reactive memory B cells for many years, even in the absence of re-immunization (boost). Therefore, the detection of plasma and memory B cells following vaccination is crucial for evaluating the durability of immunity earlier and more efficiently. The stimulation of antigen-specific abs production by the BBIBP-CorV resulted in the generation of germinal center (GC) B cells in immune organs. Therefore, FC was used to analyze total B cells, plasma cells, and GC B cells. We observed a greater proportion of B cells, GC B cells, transitional B cells, and plasmablasts in the YQZM+ Vaccine group than in the Vaccine group (Figure 8A–D, Supplementary Material S3). Additionally, the ELISpot results showed that IgG and IgM secretion levels of memory B cells were higher in mice immunized with both the vaccine and YQZM than in mice immunized with the vaccine alone (Figure 8E,F, Supplementary Material S3). These findings show that YQZM enhances vaccine-induced spike-specific Abs by promoting B cell, plasmablast proliferation, and GC reactions, indicating its immunopotentiatory potential for improving antibody responses and long-term protection.

### 2.7. Target Prediction and Analysis of PPI

**Figure 9:**
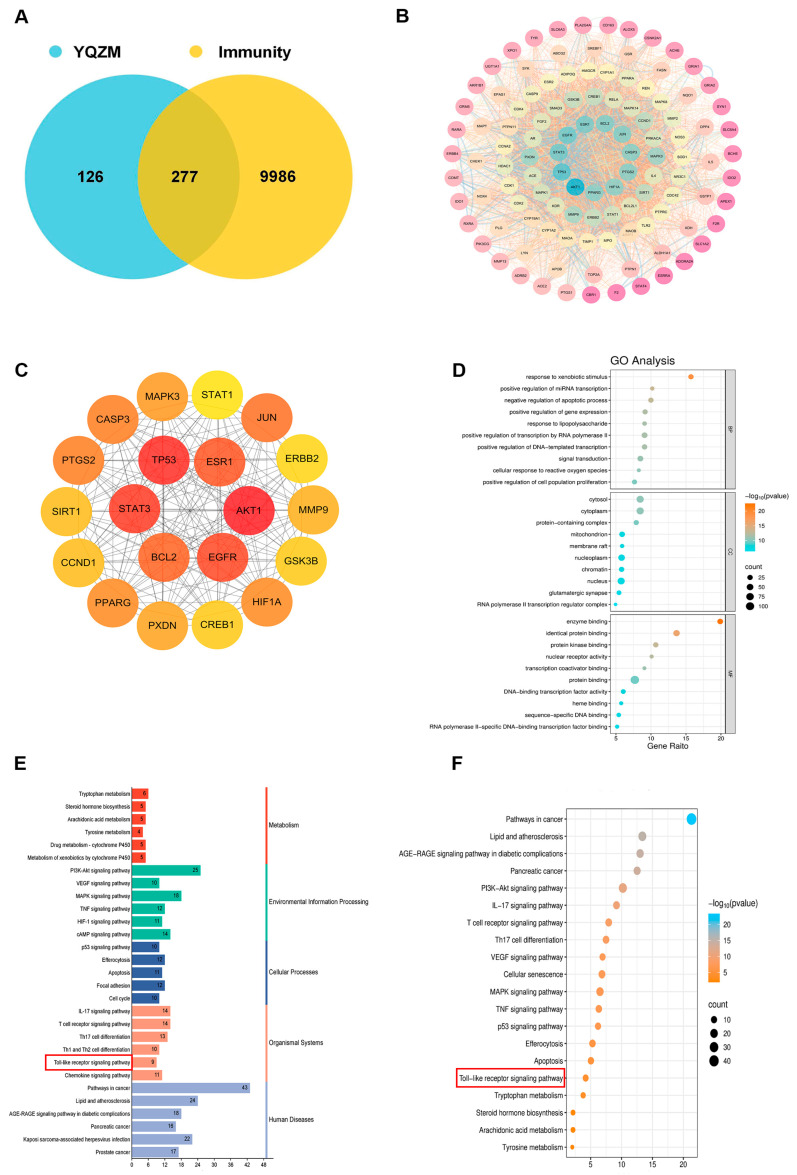
Analysis of potential YQZM action targets and pathways in enhancing immunity. (**A**) Venn diagram of potential YQZM action targets and immunity targets. (**B**) Core protein–protein interaction (PPI) network of potential YQZM action targets (114 targets). (**C**) Top 20 targets of YQZM action targets in enhancing immunity. (**D**) The top 20 significantly enriched terms of GO analysis are shown in the BP, CC, and MF categories. The *X*-axis shows the enrichment score, and the size of the circles reflects the number of genes associated with each pathway. (**E**) The secondary classification of KEGG pathways was selected, with *X*-axis representing the gene number, the left *Y*-axis representing the KEGG pathway, and the right *Y*-axis meaning secondary classification. (**F**) The top 15 significantly enriched third classification of KEGG pathways were selected, with the *X*-axis representing the enrichment score.

The targets of the 31 active components were obtained using the Swiss Target Prediction database. By cross-referencing the YQZM action targets with immunity-related disease targets, 277 overlapping target genes were identified, which represent potential key mediators of YQZM’s immune-enhancing effects (Figure 9A). The PPI network was generated by uploading the 277 potential YQZM targets associated with immunity to the STRING database, with “Homo sapiens” selected as the reference species, setting the network edge column to “Evidence”, and applying a confidence level threshold of >0.9. Using Cytoscape (v3.7.1), the target data from the STRING database were analyzed and core targets were filtered using cut-off values of CU > 0.0016, BU > 110.6235, and DU > 38.0000. This process identified 114 core targets, of which TP53, STAT3, EGFR, ESR1, BCL2, JUN, CASP3, PTGS2, HIF1A, and PPARG were ranked as the top 10 key genes (Figure 9B,C). 

GO and KEGG analyses were conducted to investigate the biological properties and signaling pathways of YQZM-immunity targets. GO analysis revealed gene enrichment for biological processes (BP), cellular components (CC), and molecular functions (MF), with the top 20 entries category (ranked by corrected *p*-values) (Figure 9D). The findings suggest that YQZM-immunity targets may play roles in processes such as “Response to xenobiotic stimulus”, “Positive regulation of miRNA transcription”, and “Negative regulation of apoptotic process”. Enriched cellular components included “Cytosol”, “Cytoplasm”, and “protein-containing complex”, while enriched molecular functions included “enzyme binding”, “identical protein binding”, and “protein kinase binding”. The second and third classification of KEGG analysis revealed that the mechanism of YQZM enhancing immunity may involve pathways including the “PI3K-Akt signaling pathway”, “MAPK signaling pathway”, T cell receptor signaling pathway”, and “Toll-like receptor (TLR) signaling pathway”, (Figure 9E,F, red boxes indicate pathways of particular biological interest). Transcriptomic and network pharmacology analyses have shown that the TLR signaling pathway is a key mechanism underlying YQZM’s immunopotentiating effects. Notably, STAT3 and STAT1 emerged among the top 20 core targets, highlighting the additional involvement of JAK-STAT signaling in YQZM’s immunomodulatory activity. These findings collectively demonstrate that YQZM enhances immune function through the coordinated regulation of both the TLR and JAK-STAT signaling cascades.

**Figure 10:**
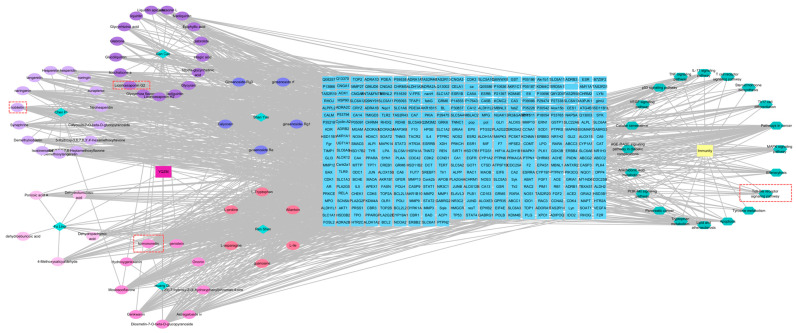
The drug-compound-target-pathway-disease network. The left-side illustrated the chemical compounds of YQZM and single botanical drug, while the right-side highlighted immunity-related functions and the top 10 KEGG pathways. The intersection gene connecting the two was displayed in the middle.

Network models were generated using Cytoscape software to map the interactions between “active components and target genes”, “active components and medicinal botanical drugs”, “target genes and pathway”, and “target genes and immunity”. This approach visually highlights the relationships between the active ingredients in YQZM and the potential targets involved in immune regulation, which underlines the top three compounds of YQZM on the left part and the Toll-like receptor signaling pathway on the right part. (Figure 10).

### 2.8. YQZM Alters the Transcriptome of dLNs from Mice Immunized with Vaccine

**Figure 11:**
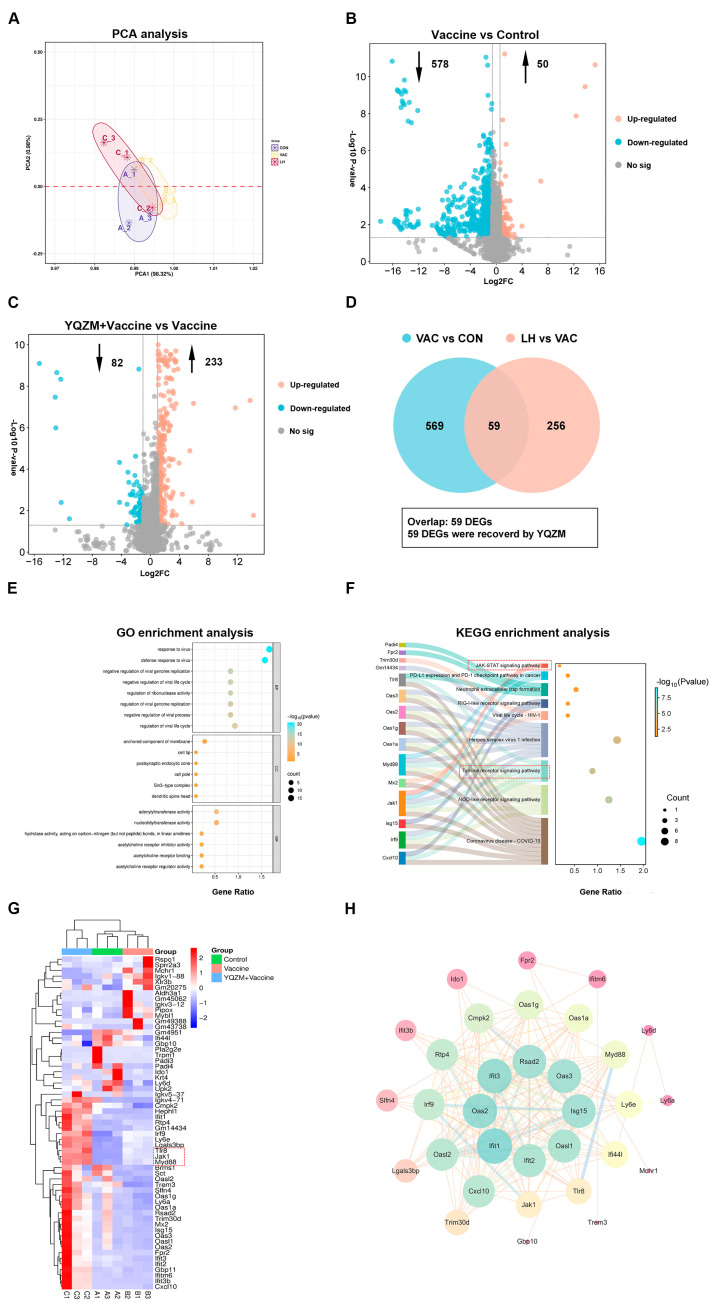
Screening for differentially expressed genes (DEGs) and functional enrichment analysis. (**A**) PCA analysis of genes in Control, Vaccine, and YQZM+ Vaccine groups. (**B**) Volcano map of DEGs between Vaccine and Control groups. (**C**) Volcano map of DEGs between YQZM+ Vaccine and Vaccine groups. (**D**) Venn diagram of DEGs between Control, Vaccine, and YQZM+ Vaccine groups. GO and KEGG enrichment analysis in Control, Vaccine, and YQZM+ Vaccine groups are shown in (**E**) and (**F**), respectively. (**G**) Heatmap of DEGs in Control, Vaccine, and YQZM+ Vaccine groups. (**H**) Protein–protein interaction network of core genes.

To further investigate the potential mechanisms by which YQZM enhances the immune response induced by the inactivated vaccine, RNA-seq was performed on draining lymph node (dLN) samples. As illustrated in Figure 11A, the PCA plot demonstrated a significant correlation among the three groups. Using the criteria |log_2_FC| > 1.2 and *p* < 0.05, 628 DEGs were identified between the Vaccine and Control groups, with 50 upregulated genes and 578 downregulated genes (Figure 11B). In addition, 315 DEGs were detected between the YQZM+ Vaccine and Vaccine groups, with 233 upregulated and 82 downregulated genes (Figure 11C). To further narrow down the potential target genes, we performed an intersection analysis of the DEGs between the Vaccine vs. Control groups and the YQZM+ Vaccine vs. Vaccine groups, and the comparisons identified 59 common genes across the three groups, as shown in the Venn diagram and cluster heatmap (Figure 11D,G). These findings suggest that the 59 DEGs play a crucial role in the immune response induced by the inactivated COVID-19 vaccine, with YQZM acting as a potential immunopotentiator by modulating the expression of these key genes. As illustrated in Figure 11F (TLR-JAK-STAT signaling pathway) and Figure 11G (associated gene expression changes), this modulation may occur through critical immune-related pathways.

As shown in Figure 11E, GO enrichment of these 59 genes yielded 246 significantly enriched terms, with the top 26 GO terms spanning BP, CC, and MF. Within the BP category, the most enriched terms were ‘response to the virus’, ‘defense response to the virus’, and ‘negative regulation of viral genome replication’. In the CC category, the most enriched terms included ‘anchored component of membrane’, ‘cell tip’, and ‘postsynaptic endocytic zone’. For MF category, the most enriched terms were ‘double-stranded RNA binding’, ‘adenylyltransferase activity’, and ‘nucleotidyltransferase activity’. The three top BP categories indicated that the immune system was activated after BBIBP-CorV vaccination. The anchored component of the membrane is a protein or structure that attaches to the cell surface, aiding membrane localization and promoting immune cell adhesion at sites of inflammation [25]. Membrane-anchored antiviral proteins, such as interferon receptors and immune cell markers, play a crucial role in activating immune responses. These proteins initiate signaling cascades that promote the production of antiviral molecules and activate immune cells, including NK cells, T lymphocytes, and macrophages [26,27]. In addition, they block viral replication by interacting with viral proteins, thereby inhibiting viral assembly and replication [28,29,30]. Collectively, these findings suggest that BBIBP-CorV vaccination activates the immune system and triggers an antiviral immune response, leading to immune cell activation upon expo-sure to viral antigens. This indicates that YQZM may enhance the antiviral immune response induced by the BBIBP-CorV, possibly through the modulation of immune cell activation and the amplification of immune responses. These mechanisms highlight the potential targets of YQZM for boosting the efficacy of BBIBP-CorV vaccination.

In KEGG enrichment analysis, the top 10 pathways are shown in Figure 11F. Specifically, these key KEGG pathways include the NOD-like receptor signaling pathway, Toll-like receptor signaling pathway, RIG-I-like receptor signaling pathway, and neutrophil extracellular trap formation, all of which are critical for antiviral immunity. Furthermore, PPI network analysis identified 10 key DEGs with high centrality that interacted with numerous proteins, suggesting their significant roles in YQZM-mediated immune modulation. These findings indicate that YQZM enhances BBIBP-CorV-induced antiviral immune responses and immune cell activation, possibly through the regulation of these critical pathways.

### 2.9. RT-qPCR and Western Blot Validation

**Figure 12:**
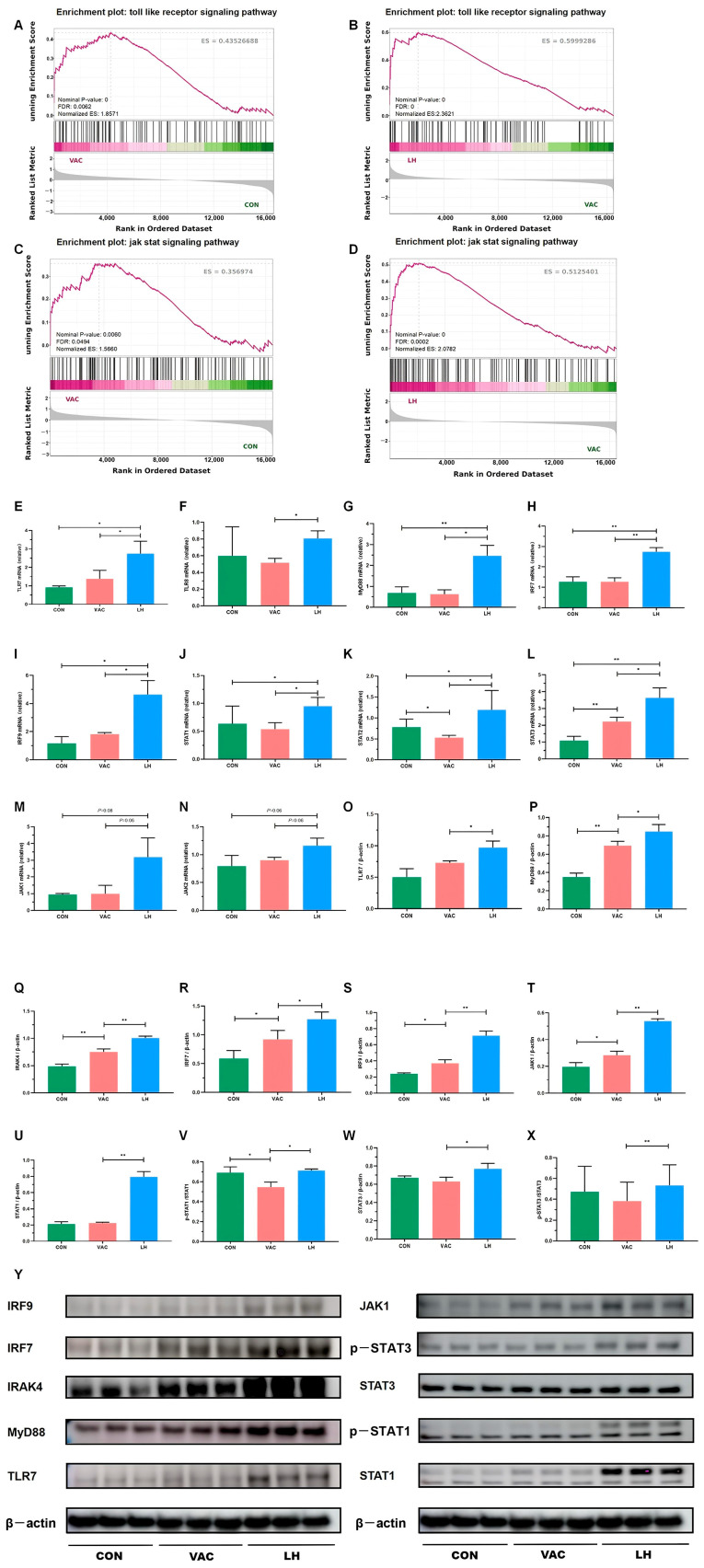
Validation of key DEGs and DEPs in the Toll-JAK-STAT signaling pathway. (**A**–**D**) GSEA analysis of the Toll-like receptor and JAK-STAT signaling pathways in the VAC vs. CON and LH vs. VAC groups. (**E**–**N**) RT-qPCR analysis showing mRNA expression levels of TLR7, TLR8, MyD88, IRF7, IRF9, STAT1, STAT2, STAT3, JAK1, and JAK2 (*n* = 3). (**O**–**X**) Western blot analysis of protein expression for TLR7, MyD88, IRAK4, IRF7, IRF9, JAK1, pSTAT1, STAT1, pSTAT3, and STAT3 in the CON, VAC, and LH groups (*n* = 3). (**Y**) WB analysis of TLR7, MyD88, IRAK4, IRF7, IRF9, JAK1, pSTAT1, STAT1, pSTAT3, and STAT3 expression across all groups. * *p* < 0.05, ** *p* < 0.01.

To further investigate the expression dynamics of genes within the Toll-like receptor and JAK-STAT signaling pathways, GSEA enrichment analysis was performed for the Control vs. Vaccine and Vaccine vs. YQZM+ Vaccine groups. As shown in Figure 12A–D, these pathways exhibited significant upregulation in the VAC group compared to the CON group. Notably, after YQZM treatment, most of the upregulated genes maintained an elevated expression trend. These results suggest that YQZM may enhance antiviral immune responses and promote immune cell activation by modulating key genes within the Toll-like receptor and JAK-STAT signaling pathways.

To validate the expression of key genes and proteins regulated by YQZM, as well as the associated proteins involved in antiviral immune responses and immune cell activation, we conducted RT-qPCR and Western blot analyses, focusing on the TLR-JAK-STAT signaling pathway, a crucial pathway that plays a significant role in antiviral immune responses, immune cell activation, and the pathogenesis of SARS-CoV-2. As shown in Figure 12E–N, RT-qPCR revealed a significant upregulation in the mRNA levels of TLR7, TLR8, MyD88, IRF7, IRF9, STAT1, STAT2, STAT3, JAK1, and JAK2 after YQZM administration. Western blotting of dLN tissues confirmed that YQZM significantly enhanced the expression of TLR7, MyD88, IRAK4, IRF7, IRF9, JAK1, pSTAT1/STAT1, and pSTAT3/STAT3 induced by the inactivated vaccine (Figure 12O–Y).

In conclusion, YQZM enhanced the antiviral immune response and immune cell activation induced by BBIBP-CorV by modulating the TLR-JAK-STAT signaling pathways, potentially improving the efficacy of the vaccine.

### 2.10. Molecular Docking Analysis of Bioactive Components with Target Proteins 

**Figure 13:**
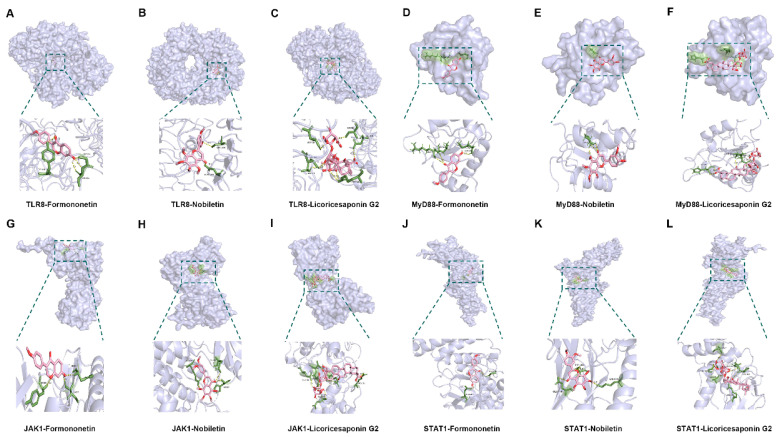
Visualization of molecular docking. (**A**–**C**) Visualization of docking results between TRL8 and Formononetin, Nobiletin, and Licoricesaponin G2. (**D**–**F**) Visualization of docking results between MyD88 and Formononetin, Nobiletin, and Licoricesaponin G2. (**G**–**I**) Visualization of docking results between JAK1 and Formononetin, Nobiletin, and Licoricesaponin G2. (**J**–**L**) Visualization of docking results between STAT1 and Formononetin, Nobiletin, and Licoricesaponin G2.

Molecular docking was performed to explore the interaction between the active components of YQZM and key nodes of the TLR-JAK-STAT signaling pathway. Three critical active components of YQZM (Formononetin, Licoricesaponin G2, Nobiletin) were chosen for molecular docking based on the degree of network pharmacology analysis, focusing on the key targets TLR8, MyD88, JAK1, and STAT1 within the TLR-JAK-STAT signaling pathway. The docking results showed good binding activity (affinity ≤ −5.0 kcal/mol) for the key components and targets, with Formononetin having the highest binding energy to TLR8, suggesting that TLR8 has the most vital binding ability. The molecular docking results for each important compound and its selected target are shown in Figure 13, indicating that the bioactive components of YQZM showed good interactions with key proteins in the TLR-JAK-STAT pathway.

## 3. Discussion

Vaccination is essential in combating infectious diseases like COVID-19 and other emerging threats. While SARS-CoV-2 vaccines offer significant benefits including rapid induction of herd immunity and high efficacy, their global implementation has revealed several limitations, such as side effects (anaphylaxis), undetermined durability of immune protection, and insufficient protective efficacy for immunocompromised populations [31,32]. These challenges highlight the critical need for new adjuvants. An ideal adjuvant should be safe, non-toxic, effective at low doses, have a clear chemical structure, and be easily metabolized and excreted by the body [33]. However, current adjuvants, such as mineral salts and Toll-like receptor agonists, have notable limitations. For example, aluminum-based adjuvants fail to induce CD8+ T cell responses and can cause side effects [34], whereas Freund’s complete adjuvant (FCA) elicits inflammation due to prolonged antigen release [35]. TCM offers several advantages as a source of adjuvants, including easy accessibility, low toxicity, and great efficacy, and they are considered natural biological regulators. In this study, we investigated a TCM-based formula, YQZM, as an immunopotentiator conjugated to the inactive BBIBP-CorV. The YQZM formula comprises six dual-purpose medicines and food with great safety, including Ren Shen, Fu Ling, Huang Qi, Shan Yao, Chen Pi, and Gan Cao. The previous research shows that high, middle, and low doses of YQZM promoted the levels of lymphocyte proliferation, enhanced paw swelling in delayed hypersensitivity assays, increased the phagocytosis rate of chicken RBC engulfed via Mø cells, and improved Abs-producing cells and HC_50_. High and medium doses of YQZM exhibited superior effects on both cellular and humoral immunity (Supplementary Material S4). Additionally, the results of acute and long-term toxicity tests suggest that YQZM is safe (Supplementary Material S5). Therefore, YQZM can be considered a potential, safe, and effective immunopotentiator.

This study evaluated the immunoenhancing effects of YQZM in mice immunized with BBIBP-CorV. The immune organ index can partly evaluate the strength of immune function [36]. Our results showed that YQZM increased the index of both the spleen and thymus in immunized mice, suggesting that it may strengthen the innate immune system. Activity of the innate immune system is crucial for effective vaccination and serves as a prerequisite for triggering subsequent adaptive immune responses [37]. After vaccination, DCs capture antigens and activate T cells, thereby initiating immune responses. Mø cells engulf the antigens and present them to T cells, contributing to cellular immunity and ensuring a strong defense against the pathogen. In the present study, co-administration of YQZM with the BBIBP-CorV significantly increased DCs and Mø compared with the BBIBP-CorV alone, indicating YQZM may potentiate the vaccine-induced immune response by promoting the function and activation of these antigen-presenting cells (APCs) [38]. The immune response of T cells and their cytokines plays a crucial role in accelerating the clearance of pathogens and producing cytokines to regulate immune responses. The activation and proliferation of CD4+ T cells results in the secretion of cytokines that regulate the activation of immune responses and activate B cells [39]. FC analysis showed that YQZM significantly increases the proportion of vaccine-induced T cells, Tfh cells, and Th1 cells, along with higher levels of IFN-γ, IL-2, and TNF-α. This finding supports the hypothesis that YQZM promotes the differentiation of T cells towards the Th1 subtype, which is closely related to the proliferation and division of lymphocytes and the maturation of DCs. Our findings suggest that YQZM exhibits multitarget immunomodulation by bidirectionally regulating APC proliferation, antigen presentation, and T cell activation/cytokine secretion to enhance the immune response.

NAbs remain the gold standard for determining vaccine efficacy, and the virus neutralization test is the classical method for intuitively displaying antibody titers. In this study, YQZM combined with the BBIBP-CorV significantly increased levels of both NAbs and anti-RBD Abs, suggesting that YQZM helps the BBIBP-CorV induce strong humoral immune responses to confer protection against SARS-CoV-2. The titers of NAbs or the level of anti-RBD are closely related to the secretory ability of B cells and the ability of Th cells to assist B cell maturation. We found that co-administration of YQZM with the BBIBP-CorV increased the number of B cells, including GC B cells, Transitional B cells, and plasmablasts. The Ab concentrations were vigorously elevated along with Tfh cells after the booster of the inactivated vaccine [40]. The production of specific antibodies induced by a vaccine can occur through two cellular pathways: the extrafollicular response and the GC reaction. The former is also called extrafollicular activation and produces an initial burst of antibodies 2–3 days after an antigenic challenge [41]. The latter, formed 1–2 weeks post antigen exposure, relies on Tfh cell assistance and could produce MB cells and long-lived plasma cells [42,43]. Additionally, ELISpot technology can quantitatively measure the number of cytokines secreted by individual cells, reflecting the degree of cell activation and function more accurately. In this present study, the spots of IL-2, IFN-γ, and TNF-α secreted from effector T cells are increased in mice with co-administration of YQZM and BBIBP-CorV. Moreover, the secretion of IgG and IgM by memory B cells also increases. The booster dose of the inactivated vaccine elicited a rapid and strong secondary response, demonstrating the establishment of long-term immune memory, including MB cells and PC-secreting high-affinity Abs [44,45]. Although antibody titers decrease over time, the generation of S-specific memory B cells mediated the recall response upon re-exposure, indicating that YQZM enhanced the ability of T and B cells to participate in the immune response [46]. In conclusion, YQZM has superior immune-enhancing effects as an adjuvant.

Most TCMs are derived from natural sources and are characterized by their therapeutic multi-component, multi-pathway, and multi-target mechanisms. Therefore, identifying the active ingredients of TCMs can aid in the analysis of their mechanisms of action and targets. Serum pharmacochemistry and UPLC-MS/MS are commonly employed to investigate and identify active compounds, which are physiological components and metabolites present in the bloodstream and are the primary contributors to the therapeutic effects of drugs [47]. In this study, a total of 52 compounds were identified in the YQZM extract through UPLC-MS/MS, and 31 compounds were identified in the serum containing YQZM, many of which were recognized for their adjuvant or immune-boosting properties. For instance, research has revealed that astragalus polysaccharide, when used as an adjuvant, promotes the proliferation of CD4+ and CD8+ T cells and significantly enhances antibody titers [48]. Pachymen is known to increase the phagocytic activity of macrophages [49]. Astragaloside A has been reported to strengthen the immune response in cyclophosphamide-induced immunosuppressed mice [50]. Flavonoids and calycosin from Astragalus regulate humoral immunity and elevate serum IgA and IgG levels [51]. Formononetin can enhance the activity and production of Th cells and has been reported to boost the immune system by promoting Th1 cell immune response [52]. Similarly, ginsenosides have been demonstrated to boost immune responses to inactivated rabies and influenza (H3N2) vaccines [53,54,55]. These findings offer valuable insights into the potential of YQZM as an immune-enhancing agent. Network pharmacological analysis of the 31 serum-detected compounds identified 114 core target proteins, with the top 20 targets containing key regulators in the JAK-STAT signaling pathway, such as STAT1 and STAT3. The KEGG enrichment analysis revealed potential pathways, including the “PI3K-Akt signaling pathway”, “MAPK signal-ing pathway”, “T cell receptor signaling pathway”, and “Toll-like receptor signaling pathway.” These findings suggest that the components of YQZM may target multiple pathways to enhance immune responses, potentially improving vaccine efficacy and strengthening overall immunity.

Transcriptomic analysis was conducted to further elucidate the underlying mechanisms of YQZM’s immunomodulatory effects. KEGG enrichment analysis showed activation of potential pathways, including the NOD-like receptor signaling pathway, Toll-like receptor signaling pathway, RIG-I-like receptor signaling pathway, and neutrophil extracellular trap formation, which is consistent with the prediction of network pharmacological analysis. Toll-like receptors (TLRs) are essential pattern recognition receptors predominantly found on innate immune cells, including DCs, NK cells, mast cells, and Møs [56]. Specifically, TLR7 is primarily located on the internal surfaces of macrophages, B cells, and plasmacytoid dendritic cells (pDCs), whereas TLR8 is found on the inner surfaces of mononuclear cells, myeloid dendritic cells (mDCs), and macrophages. Both TLR7 and TLR8 serve as pivotal signaling molecules in the innate immune system and play critical roles in the initiation of both innate and adaptive immune responses [57,58]. The genes enriched in the Toll-like receptor signaling pathway, as revealed by sequencing results, included Cxcl10, Irf9, Jak1, Myd88, and Tlr8. Notably, Myd88, and Tlr8 serve as the upstream regulators of this signaling pathway. Cxcl10, co-regulated by MyD88/NF-κB and IRF3, recruits CD8+ T cells and NK cells to infection sites, such as vaccine injection sites. Jak1 phosphorylates IRF7 in the MyD88 pathway, triggering the release of IFN-α/β (Type I interferons) [59]. Additionally, in the NOD-like receptor signaling pathway, Jak1 interacts with Irf9 to regulate interferon-stimulated genes (ISGs), such as the OAS gene family [60]. These findings suggest that YQZM may activate key genes such as MyD88 and IRF7 through the TLR signaling pathway, promoting the release of interferons, and subsequently activating the JAK-STAT signaling pathway. This mechanism highlights the potential of YQZM to enhance immune responses by modulating critical innate and adaptive immune pathways. The qPCR and Western blotting results showed similar trends, with TLR7, TLR8, MyD88, IRF7, IRF9, STAT1, STAT2, STAT3, JAK1, and JAK2 mRNA and protein expression significantly increased, indicating that YQZM likely improved the activation of the innate and adaptive immune systems through the TLR/JAK/STAT signaling pathway. Hence, we predicted the interactions between the active components of YQZM and key nodes of the TLR-JAK-STAT signaling pathway using molecular docking. The findings showed that formononetin, licoricesaponin G2, and nobiletin interacted well with key proteins in the TLR-JAK-STAT pathway.

## 4. Materials and Methods

### 4.1. Preparation and Analysis of YQZM Extracts

#### 4.1.1. Preparation of YQZM Extracts

As shown in Table 5, YQZM formulation consists of 6 different herbs, including Panax ginseng (Ren Shen, lot: 2106001), Poria cocos (Fu Ling, lot: 2004002), Astragalus membranaceus (Huang Qi, lot: 2004004), Dioscorea opposita (Shan Yao, lot: 2004001), Citri Reticulatae Pericarpium (Chen Pi, lot: 1912002), and Glycyrrhiza glabra (Gan Cao, lot: 2104004). All of the herbs were purchased from Anguo Changda Prepared Chinese Medicinal Herbs Co., Ltd. (Baoding, China). The mixture was soaked in a 10-fold volume of distilled water for 0.5 h, followed by boiling for 1 h, which was repeated twice. Then, the extract was collected and vacuum-dried. The extraction and concentration methods for each individual herb in the formulation followed the same procedure as described above. For UPLC-MS/MS analysis, the YQZM and single herb powder were weighed and mixed with extraction solvent containing internal standards. The mixture was then subjected to cryogenic grinding and extraction, followed by centrifugation to obtain the supernatant.

#### 4.1.2. Drug Administration and Sample Collection

Male and female SD rats (180~220 g, 6~8 weeks) were provided by Beijing Vitalriver Laboratory Animal Technology Co., Ltd. (Beijing, China). All animals raised under SPF (specific pathogen-free) conditions with 20~24 °C and 40~60%. The study was approved by the Experimental Animal Ethics Committee of the China Academy of Chinese Medical Sciences (No. 2021B159) and strictly followed the instruction of Organizational Guidelines and Ethics Guidelines of the Experimental Animal Ethics Committee. The total of 12 SD rats were randomly allocated into 2 groups (*n* = 6 per group): the Control group and YQZM group. The administered dose of YQZM was calculated based on the standard conversion formula for interspecies dose translation, where the rat dose (g/kg) = [6.5 g/(60 kg)] × 60 kg × 0.018/0.2 kg = 0.585 g/kg. After a 3-day acclimatization period, the rats received either YQZM extract (0.585 g/kg) or distilled water via oral administration. The experimental protocol involved twice-daily oral administration for 7 consecutive days. Two hours after the last gavage, the rats were anesthetized with Zoletil ^50^ (50 mg/kg), and blood was collected from the abdominal aorta. Serum was centrifuged, collected, and then added to extraction solvent with internal standards. The mixture was sonicated for ultrasonic extraction, centrifuged, and dried with nitrogen. Finally, the sample was reconstituted and centrifuged again, and the supernatant was transferred to an injection vial for analysis.

#### 4.1.3. UPLC-Q-Exactive Orbitrap/MS

The UHPLC-Q-Exactive Orbitrap/MS system (Thermo Fisher Scientific, Waltham, MA, USA) was employed, utilizing an ACQUITY UPLC BEH C18 column (100 × 2.1 mm, 1.7 µm) with an electrospray ionization (ESI) source. The mobile phase consisted of 2% acetonitrile–water with 0.1% formic acid (A) and acetonitrile with 0.1% formic acid (B). The column temperature was maintained at 40 °C, and the injection volume was set to 3 µL. Dual-polarity data were collected with spray voltages of +3000 V for positive mode and −3000 V for negative mode over a scan range of 70~1050 *m*/*z*. ESI source parameters included a sheath gas flow rate of 50 Arb, auxiliary gas flow rate of 13 Arb, capillary temperature of 320 °C, full MS resolution of 70,000, MS/MS resolution of 17,500, and normalized collision energies of 20, 40, and 60 eV. The raw data were analyzed using Progenesis QI v3.0 software, and the mass spectrometry data were compared with Majorbio’s custom-built Traditional Chinese Medicine Exclusive Metabolite Database (MJBIOTCM) to identify the metabolites. Partial Least Squares–Discriminant Analysis (PLS-DA) was used to find the main YQZM absorbed components. Compounds meeting dual criteria of variable importance in projection (VIP) > 1.0 and *p* < 0.05 were considered statistically significant absorbed constituents.

### 4.2. Animal Experimental Design

#### 4.2.1. Animals

A total of 48 BALB/c mice (18~22 g, 6~8 weeks, half male and female) were provided by Beijing Vitalriver Laboratory Animal Technology Co., Ltd. (Beijing, China). All animals were raised under specific pathogen-free conditions with 20~24 °C and 40~60%. This study was approved by the Experimental Animal Ethics Committee of the China Academy of Chinese Medical Sciences (No. 2021B159) and strictly followed the instruction of Organizational Guidelines and Ethics Guidelines of the Experimental Animal Ethics Committee.

#### 4.2.2. Immunization and Treatment

The mice were randomly divided into 4 groups according to body weight (*n* = 12), including the Control group, Vaccine group, YQZM group, and YQZM+ Vaccine group. The immune mice model received BBIBP-CorV via the intramuscular (*i.m.*) route; the dose for each mouse was 6.5 U/0.5 mL in the Vaccine and YQZM+ Vaccine groups at day 0 (prime) and day 14 (boost). The mice received YQZM (0.845 g/kg) by the intragastrical administration (*i.g.*) route in the YQZM and YQZM+ Vaccine groups once a day for 28 consecutive days after the prime vaccine. Meanwhile, the mice in Control group received PBS via the *i.m.* and distilled water by the *i.g.*

### 4.3. Determination of Body Weight and the Immune Organ Indexes

The body weights of the mice were recorded weekly during the experiment. At the end of the experiment, blood samples were collected via retro-orbital puncture, and the serum was separated and stored at −80 °C. Subsequently, the spleen and thymus were excised and weighed to calculate the immune organ index.

### 4.4. Antigen-Specific Antibody Measurements

The titers of anti-RBD Abs were determined using a Mouse SARS-CoV-2 Spike RBD Antibody Titer Assay Kit (Sino Biological, Chesterbrook, PA, USA). Following the manufacturer’s instructions, anti-RBD Ab titers were calculated using the following formula: anti-RBD Ab titer = (sample absorbance − blank absorbance)/(negative control absorbance − positive control absorbance).

The titers of NAbs were determined by the BJBPI (Beijing institute of biological products Co., Ltd., Beijing, China) using SARS-CoV-2 Wuhan-Hu-1 strain (GenBank: MN908947) and Vero E6 cells (CAT: 88020401). Serum, pre-diluted by 1:4 and inactivated at 56 °C for 30 min, was serially diluted and incubated with 100 TCID_50_ SARS-CoV-2. After 2 h, a 2.5×10^5^/mL Vero E6 cell suspension was added and cultured for 96 h. The neutralization titer was calculated using the Karber method based on cytopathic effects (CPEs), with a titer < 1:4 considered negative and ≥1:4 positive [6].

### 4.5. Detection of Immune Cell Subsets and Cytokine

#### 4.5.1. Preparation of Single Cell Suspensions from Spleen

Mice spleens were gently homogenized in RPMI 1640 medium using a syringe plunger and then passed through a 70 μm sterile filter. The red blood cells (RBCs) in the cell suspension were lysed and subsequently centrifuged. The resulting pellet cells were then resuspended in PBS and divided into two parts, one for flow cytometry and another for ELISpot. For ELISpot parts, the cells were counted using trypan blue and stored at −80 °C in serum-free cryopreservation solution (Dakewe Biotech, Beijing, China).

#### 4.5.2. Cell Resuscitation and In Vitro Stimulation

The single cells were cultured for 4 h and stimulated with PMA (1 μL/10^5^ cells) and monensin (2 μL/10^5^ cells) for 2 h, followed by washing to remove secretions. Cells were stimulated with the SARS-CoV-2 S protein (GenBank: MN908947, 1 μL/10^5^ cells) and monensin (2 μL/10^5^ cells) for 4 h. Then, the mixture was washed with RPMI 1640.

#### 4.5.3. Intra-/Extracellular Staining and Flow Cytometry Analysis

After incubation, cells were stained with Zombie Fixable Viability Dye (20 min, 4 °C) to discriminate viable cells. FcγIII/II receptors were blocked using anti-mouse CD16/32. Then, they were stained with fluorescent antibodies for surface markers (CD45, CD3, CD11b, CD4, CD8a, CD19, CD40, CXCR5, CD95, B220) and intracellular markers (IL-2, IL-4, IL-6, IL-10, TNF-α, IFN-γ). After fixation with 4% paraformaldehyde (30 min, 4 °C) and washing, cells were resuspended in staining buffer. Samples were analyzed on a CytoFLEX flow cytometer (Beckman Coulter, Brea, CA, USA, v3.0) using FlowJo software (TreeStar, Ashland, OR, USA, v10.8.1).

### 4.6. Detection of Effective Memory Cells

#### 4.6.1. Detection of Antigen-Specific Memory B Cells

Memory B cells were analyzed using the Mouse IgG and IgM ELISpot Basic kit (Mabtech, 3825-2A and 3885-2A). After thawing, lymphocytes were incubated in RPMI 1640 complete medium for 4 h, counted, and seeded in 96-well PVDF plates coated with mouse IgG or IgM capture antibody at 5 × 10^6^ cells/mL. Control wells included a positive control (ConA), background control (RPMI 1640), and negative control (unstimulated cells), while test wells were stimulated with S protein. Plates were incubated at 37 °C and 5% CO_2_ for 24 h. After washing, biotinylated anti-IgG or anti-IgM detection abs were added for 2 h, followed by streptavidin-alkaline phosphatase and BCIP substrate. Plates were air-dried and scanned using the Mabtech IRIS FluoroSpot/ELISpot reader. S-specific IgG or IgM-secreting memory B cells were quantified and expressed as cells per million lymphocytes and percentage of total circulating IgG or IgM-secreting memory B cells.

#### 4.6.2. Detection of Antigen-Specific Memory T Cells

S-specific T cell responses were detected using the Mouse IFN-γ/TNF-α/IL-2 FluoroSpot PLUS kit (Mabtech, FSP-414245-10). Lymphocytes (5 × 10^5^ cells) were stimulated with S protein for 24 h. Background wells contained RPMI 1640, negative wells contained cells only, and positive wells were stimulated with R848 and rmIL-2. Detection antibodies, fluorophore conjugates, and fluorescence enhancers were added sequentially. Plates were scanned and analyzed using the Mabtech IRIS FluoroSpot/ELISpot reader. Antigen-specific T cell responses were quantified by subtracting the background and negative control spots from the peptide-stimulated wells.

### 4.7. Network Pharmacology Study

These 31 chemicals identified in serum might be active ingredients of YQZM. Their molecular structures and SMILES notations were obtained from PubChem, and possible biological targets were estimated using Swiss-Target-Prediction with probability score > 0. Immunity-related targets were systematically curated from the GeneCards and OMIM databases. Through analysis of the intersection of target libraries, we identified YQZM’s potential immunomodulatory targets. The STRING database was utilized to study these targets and create a PPI network. Key nodes were identified using Cytoscape 3.7.1, with CU > 0.0016, BU > 110.6235, and DU > 38.0000. Additionally, a drug–metabolite–target–pathway–disease network was constructed using Cytoscape. Additionally, we used the DAVID database and the clusterProfiler R package to perform enrichment analysis, which helped us identify key targets and pathways through KEGG pathway and GO functional analyses.

### 4.8. Transcriptome Analysis

The total RNA of bilateral inguinal dLNs was extracted using a TRIzol reagent. RNA libraries were sequenced on the Illumina Novaseq 6000, and the data were processed on the Illumina platform. Clean data were obtained by removing low-quality sequences and rRNA contamination. The data were aligned to the Ensembl Mus musculus reference genome (GRCh38, Release 102) using HiSat2 (version 2.1.0). Gene expression levels were quantified using FPKM. Differential expression analysis was performed with DESeq2, and genes with |log_2_FC| > 1.2 and *p* < 0.05 were considered significantly differentially expressed [61]. All statistical analyses were conducted in R (version 4.0.0). Gene intensities were normalized, and hierarchical clustering was performed with the heatmap package. PCA was conducted using the metaX package, while Pearson correlation analysis was done using the cor package. Functional annotation of genes was carried out through hypergeometric enrichment analysis with KEGG, GO. The PPI were analyzed via the STRING database, with interactions having a confidence score > 0.4 included for mapping.

### 4.9. Quantitative Real-Time PCR

Total RNA was isolated from dLNs using RNA extraction solution in accordance with the manufacturer’s guidelines. Subsequently, 5 μg of the extracted RNA was reverse-transcribed into cDNA using the TransScript^®^ Uni All-in-One 1^st^ Strand cDNA Synthesis SuperMix (gDNA Removal) kit, following the provided protocol. The expression of GAPDH, IRF9, JAK1, JAK2, MyD88, STAT1, STAT2, STAT3, TLR7, and TLR8 was investigated using RT-PCR. The primer sequences of the above genes are listed in Table 6. TransStart^®^ Top Green qPCR SuperMix kit was employed to perform qRT-PCR. And the information was collected and data were analyzed using the 2^−ΔΔCT^ method. The mRNA levels of tested genes were normalized with GAPDH as an internal reference.

### 4.10. Western Blotting

Total proteins of dLNs were extracted using RIPA and then centrifuged to obtain supernatant. The protein concentration was tested using a BCA kit and through adjusting the concentration to 10 μg/μL using loading buffer and RIPA. Following separation by SDS-PAGE, proteins were transferred onto nitrocellulose membranes and blocked using 5% skim milk in TBST. The membranes were then subjected to overnight incubation with primary antibodies at 4 °C, after which they were incubated with secondary antibodies. Protein bands were visualized using the Amersham Imager 680 and quantified with ImageJ software (National Institutes of Health, Bethesda, MD, USA, v1.53).

### 4.11. Molecular Docking

The characteristic components of serum were selected according to the degree score of network pharmacology. Meanwhile, the key targets were found based on the KEGG enrichment results of RNA-seq and network pharmacology. The MOL2 molecule structures of active ingredients were obtained from the Pubchem database, and the receptor protein structures were acquired from the Protein Data Bank. PYMOL (Schrödinger, LLC, New York, NY, USA, v2.5.0) and AutoDock software (Molecular Graphics Laboratory, The Scripps Research Institute, La Jolla, CA, USA, v4.2.6) was used for dehydration, hydrogenation, molecular docking, and visualization.

### 4.12. Statistical Analysis

Paragraph Prism 8.0.2 (GraphPad Software, La Jolla, CA, USA) software was performed to analyze the data. All data were expressed as the mean ± standard deviation ($\overset{-}{x}$ ± *s*). Statistical significance was set at *p* < 0.05.

## 5. Conclusions

This study employed an integrated method, combining serum pharmacochemistry, network pharmacology, and RNA-seq, to uncover the active compounds and functional mechanisms of YQZM as an immunopotentiator with the BBIBP-CorV through the TLR-JAK-STAT signaling pathway. Our findings demonstrated that YQZM upregulates the TLR-JAK-STAT signaling pathway in DCs, promoting the maturation of DCs, thereby promoting T and B cell activation and differentiation. In conclusion, YQZM acts as a potential immunomodulator by synergistically activating the TLR-JAK-STAT pathway, bridging innate and adaptive immunity to enhance vaccine efficacy. These findings provide a scientific basis for its application in adjuvant immunotherapy.

## Figures and Tables

**Table 1:** Chemical composition of YQZM in positive ion mode (ESI+).

No.	RT/min	Name	*m*/*z*	CAS	Source
1	0.63	sucrose	365.11	57-50-1	Ren shen, Huang qi, Fu Ling, Shan yao, Chen pi, Gan cao
2	0.65	L-Proline	116.07	147-85-3	Shan yao, Fu Ling
3	0.88	Synephrine	168.10	94-07-5	Chen pi
4	1.11	L-Isoleucine	132.10	73-32-5	Fu Ling, Shan yao
5	2.84	L-Tryptophan	205.10	73-22-3	Shan yao, Fu Ling
6	4.21	Calycosin-7-O-beta-D-glucoside	447.13	20633-67-4	Chen pi
7	4.28	Liquiritin	419.13	551-15-5	Gan cao
8	4.55	Naringenin	273.08	480-41-1	Chen pi
9	4.79	Neohesperidin	611.20	13241-33-3	Chen pi
10	5.24	Isoliquiritoside	419.13	5041-81-6	Gan cao
11	5.36	Auraptenol	261.11	1221-43-8	Chen pi
12	5.46	Ononin	431.13	486-62-4	Huang qi
13	5.98	Calycosin	285.08	20575-57-9	Huang qi, Ren shen, Gan cao
14	7.07	Glucoliquiritin	581.19	93446-18-5	Gan cao
15	7.20	Moslosooflavone	299.09	3570-62-5	Gan cao, Huang qi, Chen pi
16	7.45	Gancaonin L	355.12	129145-50-2	Gan cao
17	8.02	5-O-Demethylnobiletin	389.12	2174-59-6	Chen pi
18	8.20	Isosinensetin	373.13	17290-70-9	Chen pi
19	8.56	Licoricesaponin G2	839.41	118441-84-2	Gan cao
20	8.99	Formononetin	267.07	485-72-3	Huang qi
21	9.33	6-Demethoxytangeretin	343.12	6601-66-7	Chen pi
22	9.51	Astragaloside IV	802.49	84687-43-4	Huang qi
23	9.69	Glycyrrhizic acid	823.41	1405-86-3	Gan cao
24	10.14	Nobiletin	403.14	478-01-3	Chen pi
25	10.23	Licoflavone A	323.13	61153-77-3	Gan cao
26	10.73	3’-Hydroxy-3,5,6,7,8,4′,5′-heptamethoxyflavone	433.15	5244-28-0	Chen pi
27	11.04	5-OH-HxMF	419.13	1176-88-1	Chen pi
28	11.15	Tangeretin	373.13	481-53-8	Chen pi
29	12.37	Glabrone	337.11	60008-02-8	Gan cao
30	13.43	Glycyrrhetinic acid	471.35	471-53-4	Gan cao
31	13.93	Poricoic acid A	497.33	137551-38-3	Fu Ling

**Table 2:** Chemical composition of YQZM in negative ion mode (ESI−).

No.	RT/min	Name	*m*/*z*	CAS	Source
1	0.58	L-asparagine	131.05	70-47-3	Ren shen, Shan yao, Fu Ling
2	0.64	allantoin	157.04	97-59-6	Shan yao
3	1.08	Guanosine	282.08	118-00-3	Ren shen, Shan yao, Fu Ling
4	4.22	Liquiritin apioside	549.16	74639-14-8	Gan cao
5	4.29	Liquiritin	417.12	5088-75-5	Gan cao
6	4.54	Naringin	579.17	10236-47-2	Chen pi
7	4.81	Hesperidin	609.18	520-26-3	Chen pi
8	5.35	Ginsenoside Re	991.55	52286-59-6	Ren shen
9	5.38	Ginsenoside Rg1	845.49	22427-39-0	Ren shen
10	5.60	Hydroxygenkwanin	299.06	20243-59-8	Gan cao, Huang qi
11	5.99	Genkwanin	283.06	437-64-9	Huang qi, Gan cao
12	6.23	Diosmetin-7-O-beta-D-glucopyranoside	461.11	20126-59-4	Huang qi, Gan cao
13	6.88	Genistein	269.05	446-72-0	Huang qi, Gan cao
14	7.41	Hesperetin	301.07	520-33-2	Chen pi
15	7.75	Ginsenoside Rf	845.49	52286-58-5	Ren shen
16	8.15	Ellagic acid	357.06	476-66-4	Gan cao
17	8.55	Formononetin	267.07	485-72-3	Huang qi, Gan cao
18	10.43	Licoricesaponin H2	821.40	118441-85-3	Gan cao
19	11.91	Licochalcone A	337.14	58749-22-7	Gan cao
20	12.34	Ginsenoside Rg3	829.50	14197-60-5	Ren shen
21	13.26	Poricoic acid A	497.33	137551-38-3	Fu Ling
22	13.52	Pachymic acid	483.35	29070-92-6	Fu Ling
23	13.68	3β,22β-Dihydroxy-11-oxoolean-12-en-30-oic acid γ-lactone	467.32	10401-33-9	Gan cao

**Table 3:** Chemical composition of serum containing YQZM in positive ion mode (ESI+).

No.	RT/min	Name	*m*/*z*	CAS	Source
2	0.65	L-Proline	116.07	147-85-3	Shan yao, Fu Ling
5	2.84	L-Tryptophan	205.10	73-22-3	Shan yao, Fu Ling
7	4.28	Liquiritin	419.13	551-15-5	Gan cao
8	4.55	Naringenin	273.08	480-41-1	Chen pi
9	4.79	Neohesperidin	611.20	13241-33-3	Chen pi
10	5.24	Isoliquiritoside	419.13	5041-81-6	Gan cao
12	5.46	Ononin	431.13	486-62-4	Huang qi
13	5.98	Calycosin	285.08	20575-57-9	Huang qi, Ren shen, Gan cao
15	7.20	Moslosooflavone	299.09	3570-62-5	Gan cao, Huang qi, Chen pi
17	8.02	5-O-Demethylnobiletin	389.12	2174-59-6	Chen pi
18	8.20	Isosinensetin	373.13	17290-70-9	Chen pi
19	8.56	Licoricesaponin G2	839.41	118441-84-2	Gan cao
21	9.33	6-Demethoxytangeretin	343.12	6601-66-7	Chen pi
23	9.69	Glycyrrhizic acid	823.41	1405-86-3	Gan cao
24	10.14	Nobiletin	403.14	478-01-3	Chen pi
26	10.73	3′-Hydroxy-3,5,6,7,8,4′,5′-heptamethoxyflavone	433.15	5244-28-0	Chen pi
28	11.15	Tangeretin	373.13	481-53-8	Chen pi

**Table 4:** Chemical composition of serum containing YQZM in negative ion mode (ESI−).

No.	RT/min	Name	*m*/*z*	CAS	Source
1	0.58	L-asparagine	131.05	70-47-3	Ren shen, Shan yao, Fu Ling
2	0.64	allantoin	157.04	97-59-6	Shan yao
5	4.29	Liquiritin	417.12	5088-75-5	Gan cao
7	4.81	Hesperidin	609.18	520-26-3	Chen pi
8	5.35	Ginsenoside Re	991.55	52286-59-6	Ren shen
12	6.23	Diosmetin-7-O-beta-D-glucopyranoside	461.11	20126-59-4	Huang qi, Gan cao
13	6.88	Genistein	269.05	446-72-0	Huang qi, Gan cao
14	7.41	Hesperetin	301.07	520-33-2	Chen pi
15	7.75	Ginsenoside Rf	845.49	52286-58-5	Ren shen
16	8.15	Ellagic acid	357.06	476-66-4	Gan cao
17	8.55	Formononetin	267.07	485-72-3	Huang qi, Gan cao
18	10.43	Licoricesaponin H2	821.40	118441-85-3	Gan cao
20	12.34	Ginsenoside Rg3	829.50	14197-60-5	Ren shen
22	13.52	Pachymic acid	483.35	29070-92-6	Fu Ling

**Table 5:** Composition of YQZM.

Chinese Name	Latin Name	Medicinal Part	Source	Weights/g
Ren Shen	Panax ginseng	Radix and rhizome	*Panax ginseng* C. A. Mey.	10
Huang Qi	Astragalus membranaceus	Radix	*Astragalus membranaceus* (Fisch.) Bge.var.*mongholicus* (Bge.) *Hsiao*	15
Chen Pi	Citri Reticulatae Pericarpium	Peel	*Citrus reticulata Blanco*	15
Fu Ling	Poria cocos	Sclerotium	*Poria cocos (Schw.) Wolf*	9
Shan Yao	Dioscorea opposita	Rhizome	*Dioscorea polystachya Turcz.*	9
Gan Cao	Glycyrrhiza glabra	Radix and rhizome	*Glycyrrhiza uralensis Fisch.*	7

**Table 6:** Primer sequence.

Name	Upstream (5′-3′)	Downstream (5′-3′)
GAPDH	AAATTCAACGGCACAGTCAA	TAGACTCCACGACATACTCAGCA
IRF7	GCGTACCCTGGAAGCATTTC	GCACAGCGGAAGTTGGTCT
IRF9	CCTCAGGCAAAGTACGCTG	GGGGTGTCCTATGTCCCCA
JAK1	CTCTGGTATGCTCCAAATCG	TGTCCATCCTGCTCGGTC
JAK2	GAGCTACTGAAGAACAACGG	TGAAAGAGGGACGTTGGTTGA
MyD88	GGCCCCGGTCTCCTCCACA	GCCACCTGTGTCCGCACGTT
STAT1	CTATGATGTCTCGTTTGCG	CTTTTCCGTATGTTGTGCTG
STAT2	CCAGCTTTACTCGCACAGC	AGCCTTGGAATCATCACTCCC
STAT3	AGCAGCAGGAAAATGGCTCCTCCAG	GTTCACTCAAAGGCGAGGGTTGTGG
TLR7	AAGGGGTATCAGCGTCTA	GTATGCTCTGGGAAAGGT
TLR8	TCGTCTTGACCGTTTGTGGAATG	CCATTTGGGATTTGTTGAAGGTTATT

## Data Availability

DAS to data is contained within the article.

## Supplementary Materials

The following supporting information can be downloaded at: https://www.mdpi.com/article/10.3390/ph18060802/s1. Supplementary Material S1: Flow cytometry analysis of innate immune cell subsets. Supplementary Material S2: Flow cytometry and ELISpot profiling of T cell subsets. Supplementary Material S3: Flow cytometry and ELISpot characterization of B cell subsets. Supplementary Material S4: Immunomodulatory effects of YQZM in normal mice. Supplementary Material S5: Safety assessment of YQZM treatment.

## Abbreviations

The following abbreviations are used in this manuscript:

ASCs	Plasmablast cells
COVID-19	corona virus disease
DC	Dendritic cell
GC B	germinal center B cell
Mø	macrophage cell
Nabs	neutralizing antibodies
NK	Natural killer cell
TCM	Traditional Chinese Medicine
Tfh cells	follicular helper T cells
Th1 cells	T helper 1 cells
Tran B	Transitional B cell
UPLC-ESI/MS	Ultra Performance liquid chromatography–electrospray ionization/mass spectrometry system
YQZM	Yiqi Zengmian
