# Supplementary material for: Mechanistic Exploration of Yiqi Zengmian in Regulating the Microenvironment as an Immunopotentiator with the Beijing Bio-Institute of Biological Products Coronavirus Vaccine Based on Transcriptomics and Integrated Serum Pharmacochemistry
Source: Pharmaceuticals (Basel). 2025 May 27;18(6):802. doi: 10.3390/ph18060802 (PMC12195801; doi:10.3390/ph18060802)
Supplement: Supplementary file 1 [file pharmaceuticals-18-00802-s001.zip › pharmaceuticals-3628216-supplementary.pdf]

Supplementary material

1. S1-The figures of flow cytometry with innate immune cell subset

YQZM improved the proportion of innate immune cell subsets, including the natural killer cells (NK), dendritic cells (DCs), and macrophages (Mø). The flow cytometry figures were shown in Figure S1A-C.

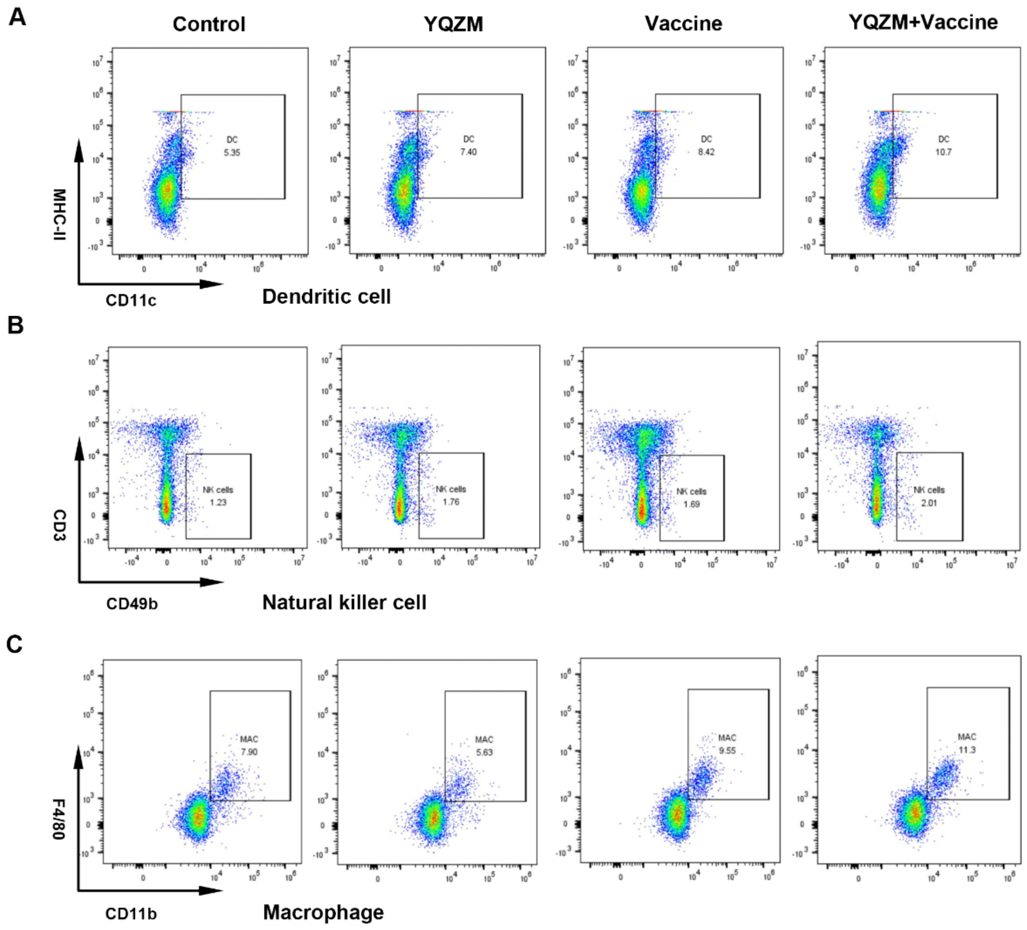

Figure S1. The innate immune cell subsets in immune mice with BBIBP-CorV. **(A)** The percentage of DCs in $CD45^+$ cells ($CD45^+$ $CD11c^+$ MHC-II^+). **(B)** The percentage of NK cells in $CD45^+$ cells ($CD45^+$ $CD49b^+$ $CD3^-$). **(C)** The percentage of Mø in $CD45^+$ cells ($CD45^+$ $CD11b^+$ F4/80^+).

2. S2-The figures of flow cytometry and ELISpot with T cell subset

YQZM increased the release of IL-2 in S-specific $CD3^+$ T cells, IL-2, IFN-γ in S-specific $CD4^+$ T cells, and IL-2, IL-4, TNF-α in S-specific $CD8^+$ T cells. The flow cytometry figures were shown in Figure S2A-F. Meanwhile, the proportion of T cell, Tfh cell (Follicular helper T cell) and Th1 cell (T helper cell 1) was higher in YQZM+ Vaccine group than Vaccine group (Figure S3A-C). The findings of ELISpot showed that the IFN-γ, TNF-α, IL-2 secreted by memory T cells were significantly enhanced in

the YQZM+ Vaccine group compared to the Vaccine group.

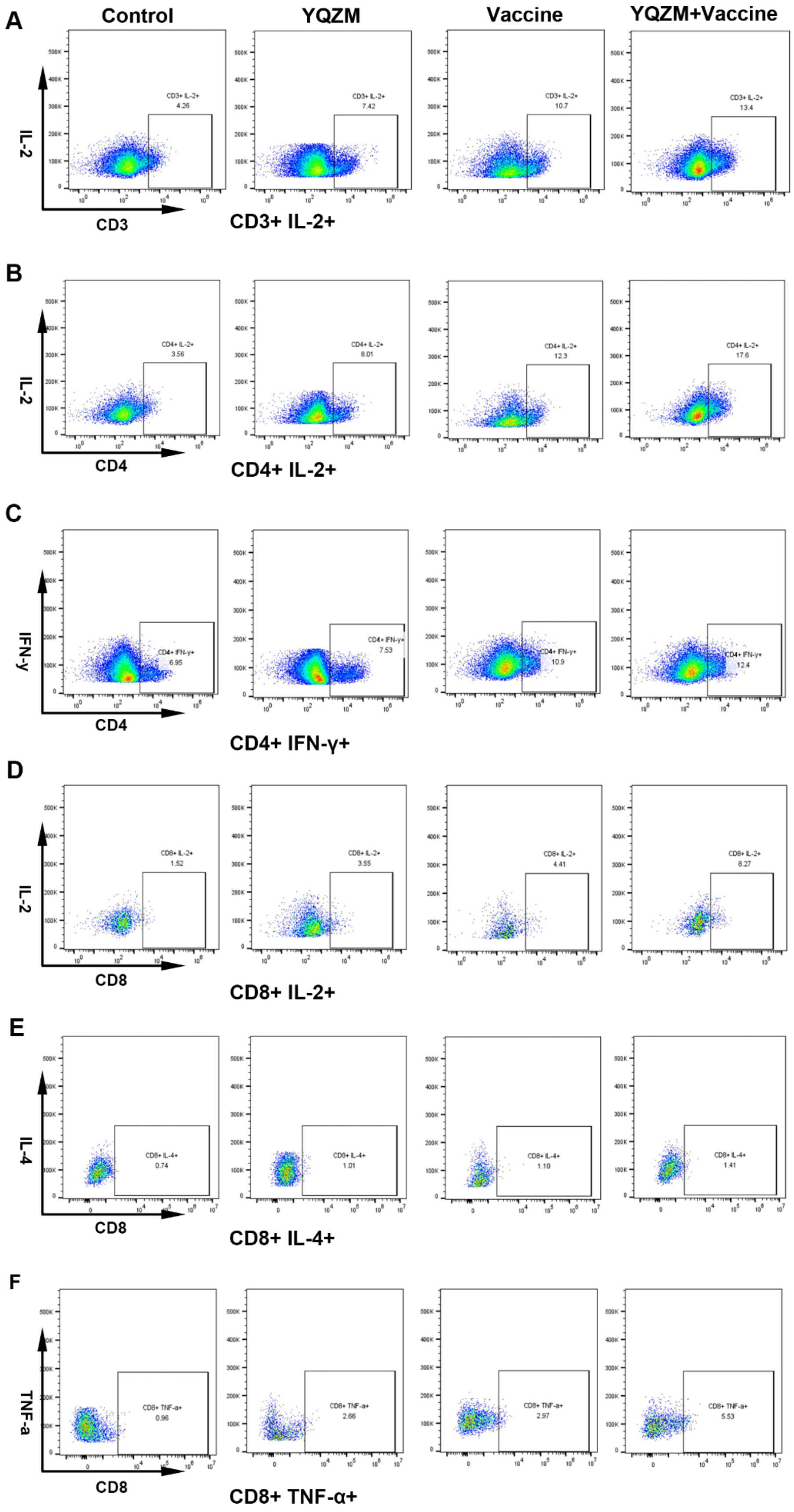

Figure S2 The levels of S- specific cytokines secreted by T cells. **(A)** The S- specific IL-2 secreted by T cells ($CD3^+$). **(B)** The S- specific IL-2 secreted by $CD4^+$ T cells. **(C)** The S- specific IFN-γ secreted by $CD4^+$ T cells. **(D)** The S- specific IL-2 secreted by $CD8^+$ T cells. **(E)** The S- specific IL-4 secreted by $CD8^+$ T cells. **(F)** The S- specific TNF-α secreted by $CD8^+$ T cells

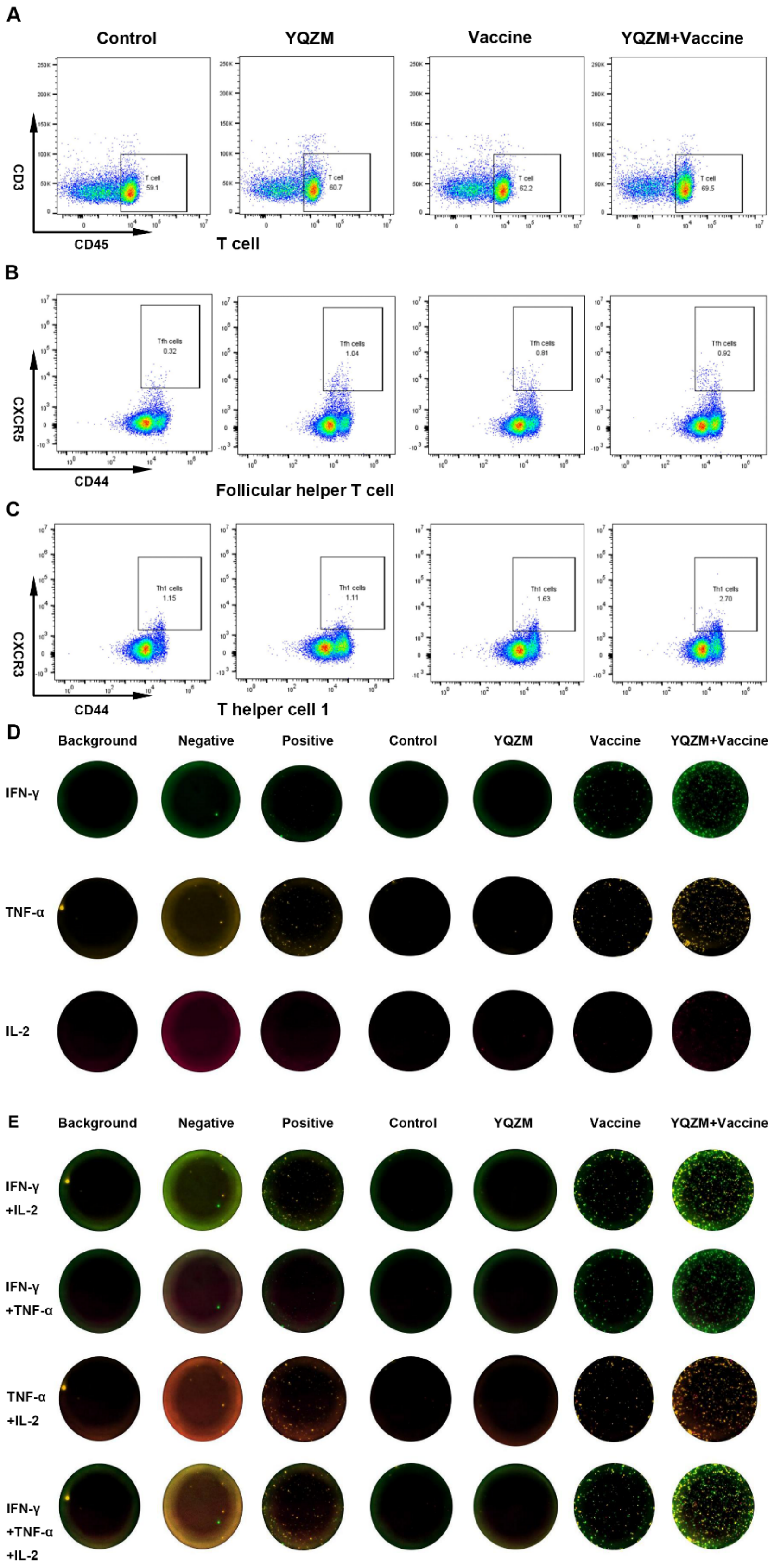

Figure S3 The subset of T cells and the S-specific IFN-γ, TNF-α and IL-2 secreted by memory T cells. **(A)** The percentage of T cells in $CD45^+$ cells ($CD45^+$ $CD3^+$). **(B)** The

percentage of Tfh cells in T cells ($CD3^+$ $CD44^+$ $CXCR5^+$). **(C)** The percentage of Th1 cells in T cells ($CD3^+$ $CD44^+$ $CXCR3^+$). **(D)** The results of ELISpot assay showed the levels of S-specific IL-2, IFN-γ and TNF-α produced by memory T cells.. **(E)** The multiple fluorescent spots of memory T cells.

3. S3-The figures of flow cytometry and ELISpot with B cell subset

The proportion of B cell, GC B cell, Transitional B cell, Plasmablast cell was higher in YQZM+ Vaccine group than Vaccine group (Figure S4A-D). And YQZM increased the release of S-specific IgG and IgM of memory B cells (Figure S4E).

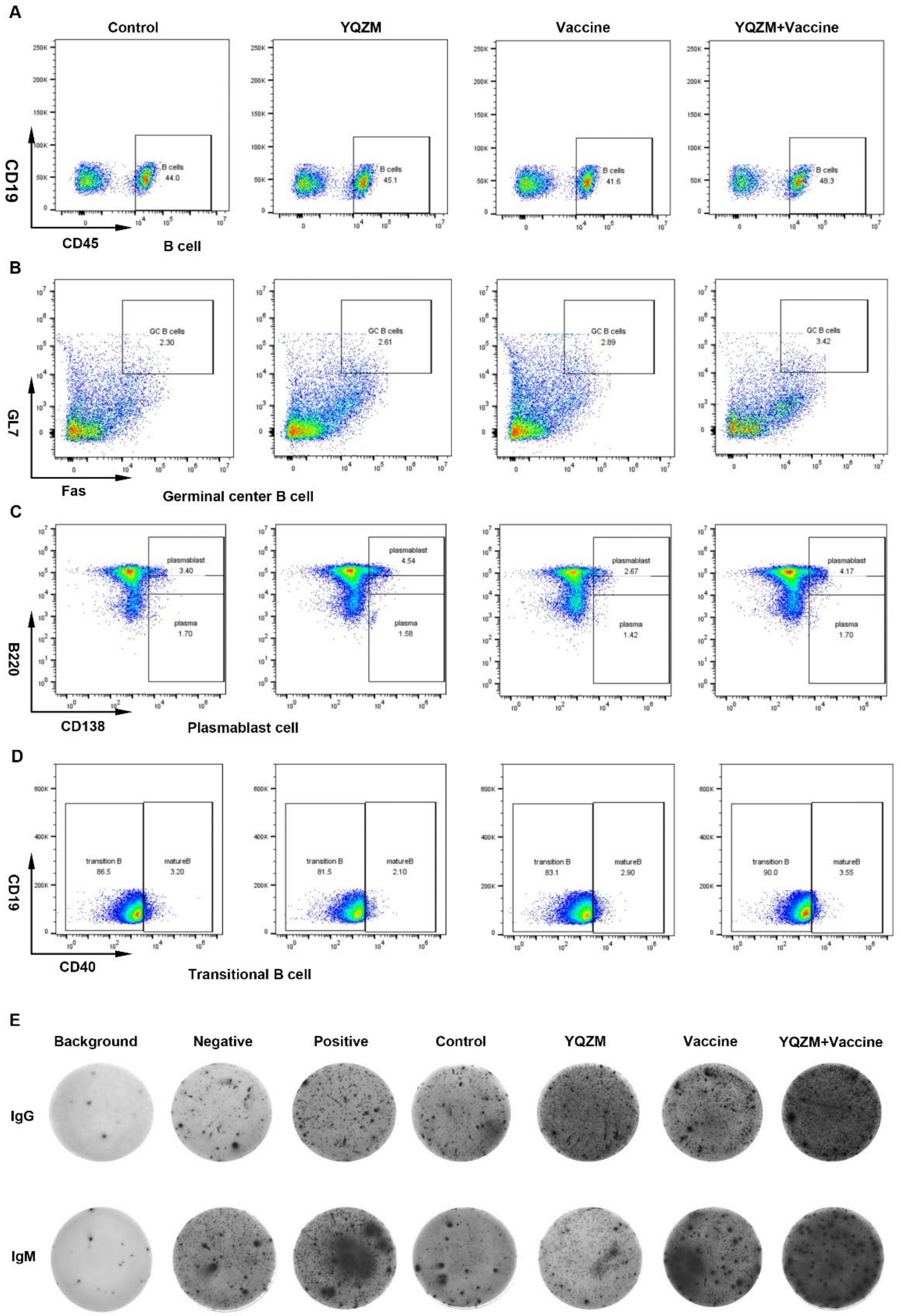

Figure S4 The subset of B cells and immunoglobulins secreted by memory B cells in immune mice with BBIBP-CorV. The percentage of B cells ($CD45^+$ $CD19^+$), germinal center B cells (Fas^+ $GL7^+$), plasmablast cells ($CD138^+$ $B220^+$), and transitional B cells ($CD19^+$ $CD40^-$) were shown in **(A), (B), (C), (D)**, respectively. The results of ELISpot of S-specific IgG and IgM of memory B cells were shown in **(E)**.

4. S4- YQZM enhanced the immune function of normal mice

1.1 Animal experimental design

Male and female BALB/c mice (6~8 weeks, 18~22 g) were provided by Beijing Vitalriver Laboratory Animal Technology Co., Ltd. (Beijing, China). All animals raised under SPF conditions with 20~24 °C and 40~60 %. After a 3-day acclimatization, mice were randomly divided into 16 groups (n=12 per group), with every 4 groups forming an experimental batch (control and high-, medium-, low-dose of YQZM groups). Batch 1 assessed organ-to-body weight ratio, delayed-type hypersensitivity (DTH), HC_{50}, and antibody-producing cells; Batch 2 performed carbon clearance assays; Batch 3 evaluated ConA-induced lymphocyte proliferation, NK cell activity, and splenic immune cell subsets; Batch 4 measured peritoneal macrophage phagocytosis of chicken red blood cells. The administered dose of YQZM was calculated based on the standard conversion formula for interspecies dose translation, where the mouse dose (g/kg) = [6.5 g/(60 kg)] × 60 kg × 0.0026/0.02 kg = 0.845 g /kg. The high-, medium-, and low-dose of mice were 2-fold, 1-fold and 0.5-fold of equivalent dose for mice, respectively. Therefore, YQZM groups received 1.690, 0.845, or 0.423 g/kg orally, while the control group received purified water, all administered daily for 28 days at 20 mL/kg.

4.2 Cellular Immune Function Assays

Spleen Lymphocyte Proliferation and Transformation Test: After 28 days of continuous administration, mice spleens were harvested, washed with Hank's solution, and passed through a sterile 70 μm cell sieve. The tissue was gently ground with a syringe core while adding Hank's solution. The cell suspension was centrifuged at 400 g for 10 minutes. The supernatant was discarded, and the cell pellet was resuspended in 2 mL of red blood cell lysis buffer for 3 minutes. The lysis reaction was stopped by adding 10 mL of Hank's solution, followed by centrifugation at 400 g for 10 minutes. The pellet was resuspended in RPMI 1640 medium, and live cell counting was performed to ensure >95% viability. Splenic lymphocytes were seeded into a 96-well plate (100 μL/well). For the experimental wells, 10 μL/well of Con A was added, while blank control wells received 10 μL/well of PBS. Cells were cultured at 37°C with 5% CO2 for 24 hours. After incubation, CCK-8 solution was added, and the reaction proceeded for 2 hours. Absorbance was measured at 450 nm, and the lymphocyte transformation degree was calculated as:

Lymphocyte Transformation Degree = OD (experimental well)/OD (blank control well).

Delayed-Type Hypersensitivity (DTH) Test: On day 24 of administration, mice were intraperitoneally injected with 2% SRBC (200 μL, ~1 × 10^8 cells) daily for 4 days to induce sensitization. On day 28, the thickness of the left hind paw was measured three times using a vernier caliper, and the average was recorded as the baseline value.

Subsequently, 20% SRBC (20 μL) was injected subcutaneously into the left hind footpad. After 24 hours, the paw thickness was measured again three times, and the average was recorded as the experimental value. The degree of swelling was calculated as:

DTH Degree = Experimental Value - Baseline Value.

4.3 Humoral Immune Function Assays

Antibody-Producing Cell Detection: SRBC-immunized mice were prepared as section 1.2, and a single-cell suspension of splenocytes was obtained. The cell concentration was adjusted to 5×10^6/mL. The 30 mm sterile Petri dish was coated with 0.5% agarose, air-dried in a clean bench, and set aside. A mixture of 20 μL splenic lymphocyte suspension and 10% SRBC was spread evenly on the dish, followed by the addition of 0.5 mL culture medium (1% agarose diluted with SA buffer mixed with 2% Hank's solution). The dish was incubated at 37°C with 5% CO2 for 1.5 hours. Guinea pig serum complement was added, and incubation continued for another 1.5 hours. The number of hemolytic plaques was counted, and the number of antibody-producing cells was calculated as:

Antibody-Producing Cells = Number of Hemolytic Plaques/Total Number of Splenic Cells.

Serum Hemolysin Determination: SRBC-immunized mice were prepared as described i as section 1.2. Blood was collected from the orbital sinus 4 days post-immunization, and serum was isolated and diluted 300-fold with SA buffer. A 1.0 mL aliquot of the diluted serum was added to a test tube, followed by 0.5 mL of 10% SRBC and 1.0 mL of complement diluent. A blank control tube (without SRBC-immunized mouse serum) was also prepared. Tubes were incubated in a 37°C water bath for 15 minutes, followed by an ice bath to terminate the reaction. After centrifugation, 1.0 mL of supernatant was mixed with 3 mL of Drabkin's reagent. A positive control tube containing 0.25 mL of 10% SRBC and 3.75 mL of Drabkin's reagent was also prepared. After 10 minutes, absorbance (OD) was measured at 540 nm, and the hemolysin level was expressed as the half-hemolysis value (HC50), calculated as:

HC_{50} = (OD Test Tube - OD Blank Control Tube)/(OD Positive Control Tube - OD Blank Control Tube) × Sample Dilution Factor.

4.4 Monocyte-Macrophage Phagocytic Function Assays

Carbon Clearance Experiment: After 28 days of continuous administration, diluted Indian ink was injected intravenously (0.05 mL/10 g) into the tail vein of mice. Blood samples (20 μL) were collected from the orbital sinus at 2 and 10 minutes post-injection and added to test tubes containing 2.0 mL of 0.1% Na_2CO_3 solution. After mixing, absorbance (OD) was measured at 600 nm using 0.1% Na_2CO_3 as a blank

control. Mice were then euthanized by cervical dislocation, and the liver and spleen were weighed. The phagocytic index (α) and carbon clearance capacity (K) were calculated as:

$K = (\log OD_1 - \log OD_2)/(t_2 - t_1)$

α = Body Weight/(Liver Weight + Spleen Weight) × K^ (1/3).

Peritoneal Macrophage Phagocytosis of Chicken Red Blood Cells: At the end of the experiment, mice were injected intraperitoneally with 1 mL of 20% chicken red blood cell suspension. After 30 seconds, mice were euthanized by cervical dislocation, and 2 mL of physiological saline was injected into the peritoneal cavity. The abdomen was gently massaged 20 times, and 1 mL of peritoneal wash solution was collected and dropped onto two glass slides. Slides were placed in a culture dish lined with wet gauze and incubated at 37°C with 5% CO_2 for 30 minutes. After rinsing, air-drying, fixing, and staining with Giemsa phosphate buffer, slides were rinsed again and air-dried. Phagocytic rate and phagocytic index were calculated under a microscope as:

Phagocytic Rate (%) = (Number of Macrophages Engulfing Chicken Red Blood Cells/Total Number of Macrophages) × 100%

Phagocytic Index = Total Number of Chicken Red Blood Cells Engulfed/Total Number of Macrophages.

4.5 NK Cell Activity Assay

At the end of the experiment, mouse spleens were harvested, and a single-cell suspension of splenocytes was prepared as described in Section 2.3.2. The cell concentration was adjusted to 1×10^6/mL. A U-shaped 96-well cell culture plate was used, with 100 μL of NK cells (effector cells) added to each well, followed by 50 μL of YAC-1 cells (target cells, 1×10^6/mL). A negative control well (100 μL culture medium + 50 μL target cells) and a positive control well (2.5% Triton X-100 solution + target cells) were also prepared. The plate was incubated at 37°C with 5% CO_2 for 4 hours. The supernatant was collected by centrifugation and mixed with LDH substrate solution. Absorbance was measured at 490 nm, and NK cell killing ability was calculated as:

NK Cell Killing Ability (%) = (OD Experimental Well - OD Negative Control Well)/(OD Positive Control Well - OD Negative Control Well) × 100%.

1.6 Results

The high, medium, and low dosage of YQZM could enhance the innate immunity and adaptive immunity (1.690, 0.845, 0.423 g/kg); Moreover, the high, medium dosage of YQZM could increase the non-specific immunity (1.690, 0.845 g/kg).

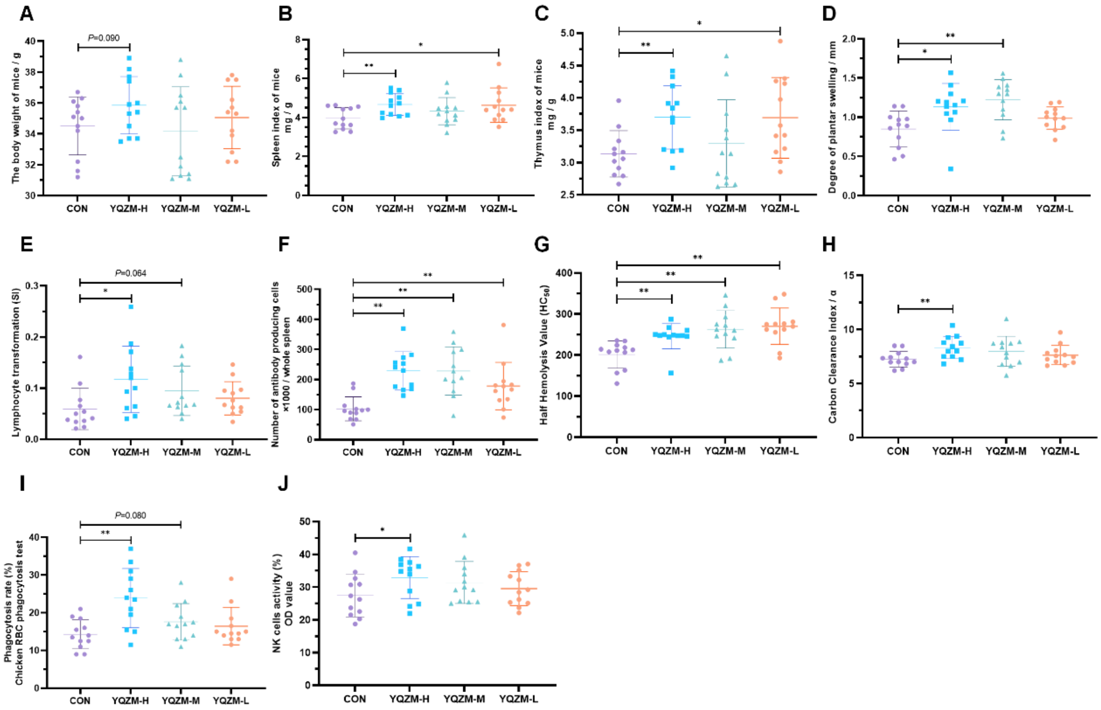

Figure S4 (A) The body weight of mice in each group. **(B-C)** The spleen index and thymus index respectively. **(D)** The degree of plantar swelling. **(E)** Lymphocyte transformation (SI). **(F)** The number of antibody-producing cells. **(G)** HC_{50}. **(H)** Carbon clearance rate. **(I)** Phagocytic index (α). **(J)** NK cells activity rate.

5. S5-Safety evaluation of YQZM

2.1 Acute toxicity experiment

In the preliminary experiment, the maximum administration dosage was set as the highest dosage. The maximum concentration of YQZM was 0.57 g/mL, the maximum administration volume for mice was 40 mL/kg, and maximum daily oral gavage frequency was 2 times. Thus, the maximum dosage for mice was 45.60 g/kg (0.57 g/mL × 40 mL/kg × 2 = 45.60 g/kg). The other three doses-36.48 g, 29.18 g, and 23.35 g /kg-were determined by sequentially reducing the maximum dosage by a factor of 1.25.

Based on preliminary experimental results, the maximum dosage (45.60 g/kg) was selected as the oral gavage dose for mice. 40 ICR mice were randomly divided into groups according to body weight, with 20 mice per group. Each group received the corresponding dose of YQZM by orally gavage twice daily for 14 consecutive days.

The results showed no toxic reactions or adverse effects in any group during the administration period. YQZM Formula was orally administered to mice twice daily at a dose of 45.60 g/kg and a volume of 40 mL/kg. No animal deaths or adverse reactions were observed.

2.2 Long term toxicity experiment

For long-term toxicity experiments, the low, medium, and high doses were set at 15, 30, and 60 times the equivalent clinical dose, respectively. Thus, the high, medium, and low doses of YQZM were 35.10 g, 17.55 g, and 8.78 g /kg, respectively. SPF-grade SD rats (half male, half female, 180~220 g) were randomly divided into a normal control group and high-, medium-, and low-dose groups, with 12 rats per group. Each group received the corresponding dose orally once daily for 90 consecutive days. After administration, a subset of rats from each group was euthanized for autopsy, while the remaining animals entered a 28-day recovery period without further treatment. This result presents only a portion of the experimental data. Additional tests, including ophthalmic examination, hematological analysis, and pathological assessment, can be provided separately upon request.

2.3 Results

The findings showed that YQZM had great safety.

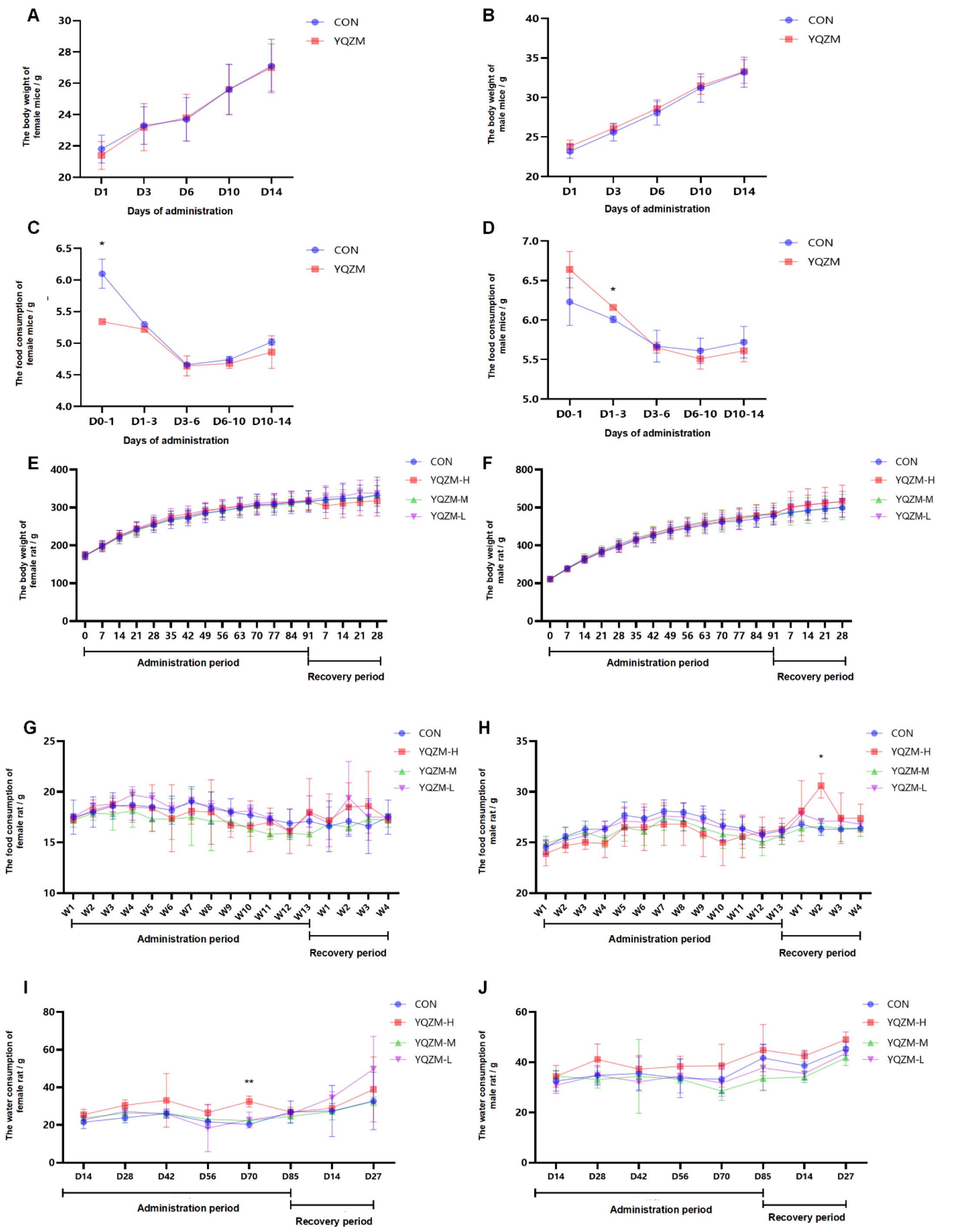

Figure S5 The changes in body weight, food intake, and water intake during the safety evaluation period. **(A-B)** The body weight of female and male mice during acute toxicity experiments. **(C-D)** The food intake of female and male mice during acute toxicity experiments. **(E-F)** The body weight of female and male rats during long term toxicity experiment. **(G-H)** The food intake of female and male rats during long term toxicity experiment. **(I-J)** The water intake of female and male rats during long term toxicity experiment.
